# Epigenetic Control of an Auxiliary Subunit of Voltage-Gated Sodium Channels Regulates the Strength of Drug-Cue Associations and Relapse-Like Cocaine Seeking

**DOI:** 10.1016/j.biopsych.2025.01.027

**Published:** 2025-02-07

**Authors:** Daniel J. Wood, Evgeny Tsvetkov, Susana Comte-Walters, Colin L. Welsh, Michelle Bloyd, Timothy G. Wood, Rose Marie Akiki, Ethan M. Anderson, Rachel D. Penrod, Lalima K. Madan, Lauren E. Ball, Makoto Taniguchi, Christopher W. Cowan

**Affiliations:** Department of Neuroscience, Medical University of South Carolina, Charleston, South Carolina (DJW, ET, MB, TGW, RMA, EMA, RDP, MT, CWC); Medical Scientist Training Program, Medical University of South Carolina, Charleston, South Carolina (DJW, MB, RMA); and Department of Pharmacology and Immunology, Medical University of South Carolina, Charleston, South Carolina (SC-W, CLW, LKM, LEB).

## Abstract

**BACKGROUND:**

Repeated use of addictive drugs produces long-lasting and prepotent drug-cue associations that increase vulnerability for relapse in individuals with a substance use disorder. Epigenetic factors, such as HDAC5 (histone deacetylase 5), play a key role in regulating the formation of drug-cue associations, but the underlying mechanisms remain unclear.

**METHODS:**

We used a combination of molecular biology, cultured cells, tandem mass spectrometry, deacetylase activity measurements, co-immunoprecipitation, and molecular dynamics simulations to assess HDAC5 structure-activity relationships. In male and female Long Evans rats, we used viral-mediated expression of HDAC5 mutants in the nucleus accumbens (NAc) to test effects on cocaine intravenous self-administration and cue-reinstated cocaine seeking. We also used in silico analysis of single-nucleus RNA sequencing data, quantitative reverse transcriptase–polymerase chain reaction, viral-mediated expression of Scn4b short hairpin RNA, patch-clamp electrophysiology, and rat cocaine or sucrose SA to assess Scn4b’s effects on NAc intrinsic excitability and cued reward seeking.

**RESULTS:**

We discovered that 2 conserved cysteines located near HDAC5’s catalytic domain were required for its intrinsic deacetylase activity and that HDAC5’s deacetylase activity was required in NAc medium spiny neurons (MSNs) to limit relapse-like cue-reinstated cocaine seeking. Moreover, we found that HDAC5 limited cocaine-seeking, but not sucrose-seeking, behavior by reducing NAc MSN intrinsic excitability through the deacetylase-dependent repression of *Scn4b*, which codes for an auxiliary subunit of voltage-gated sodium channels.

**CONCLUSIONS:**

Our findings suggest that HDAC5’s control of NAc *Scn4b* expression governs the formation of cocaine-cue, but not sucrose-cue, associations through modulation of NAc MSN intrinsic excitability and drug-induced NAc plasticity mechanisms.

Substance use disorder (SUD) is characterized by compulsive drug seeking and use despite adverse consequences (1), and relapse affects a large proportion of individuals with SUD who attempt to discontinue drug use (2,3). One major contributor to relapse risk is the presence of enduring associations between the primary rewarding effects of the drug and cues in the drug-use environment. Understanding how these drug-cue associations are formed and maintained over time could inform the development of relapse-targeted therapies.

The nucleus accumbens (NAc) is a basal forebrain region comprising predominantly medium spiny neurons (MSNs) (4), and it is an essential component of brain circuits that mediate drug-seeking behaviors (5). Epigenetic mechanisms within NAc MSNs modulate the formation, strength, and stability of many SUD-related behaviors, including drug-cue/context associations (6–8). The epigenetic enzyme HDAC5 (histone deacetylase 5) is a transcriptional repressor regulated by cocaine, ethanol, and heroin (9–13), and reduced NAc HDAC5 messenger RNA (mRNA) levels have been detected in human postmortem samples from patients with opioid use disorder (14). Like other class IIa HDACs, HDAC5 shuttles between the nucleus and the cytoplasm, and its steady-state localization is governed by activity-dependent phosphorylation (10,15–17). In rat NAc neurons, HDAC5 suppresses future cue-induced drug seeking without affecting sucrose seeking, while reduction of HDAC5 augments future cue-induced drug seeking (9,11). HDAC5 appears to limit future drug seeking by suppressing the strength of drug-cue associations formed during active drug use, but how HDAC5 limits drug-cue memories and drug seeking remains unclear.

Class IIa HDACs exhibit reduced intrinsic HDAC activity compared with their class I counterparts (18–20). HDAC5 can bind to the class I HDAC, HDAC3, which has also been linked to the regulation of SUD-related behavior (21–23), suggesting that HDAC5’s intrinsic deacetylase activity may be dispensable for its function and that a HDAC5:HDAC3 complex may be critical for regulating cued drug seeking.

HDAC5 represses the immediate early gene *Npas4*, and reduction of pan-neuronal NPAS4 in the NAc has been shown to mimic HDAC5’s ability to block noncontingent cocaine reward-context learning but not cue-reinstated cocaine seeking (11). HDAC5’s suppression of cue-reinstated drug seeking has been associated with a robust decrease in NAc MSN intrinsic excitability and the downregulation of several genes linked to transmembrane cation transport (9). One of these genes, *Scn4b*, encodes an auxiliary subunit of voltage-gated sodium channels (24–26). SCN4B is highly expressed in the striatum (27), where it is enriched in the unmyelinated projections of MSNs (28) and is well positioned to regulate NAc MSN excitability via its reported function as an open-channel blocker (28–33). In this study, we found that HDAC5 limits cued cocaine seeking through an intrinsic deacetylase activity–dependent negative regulation of *Scn4b* and NAc MSN intrinsic excitability.

## METHODS AND MATERIALS

Detailed methods are included in the Supplement. See Key Resources Table for details on materials/reagents.

### Animals and Animal Care

All behavioral experiments were performed during the dark cycle and were approved by the Medical University of South Carolina Institutional Animal Care and Use Committee. All procedures were conducted in accordance with the National Institutes of Health and National Research Council guidelines.

### Recombinant Plasmids and Viral Vectors

N-terminal 3XFLAG-tagged HDAC5 point mutants were created via a polymerase chain reaction–based strategy using the previously described neurotropic AAV2 (adeno-associated vector serotype 2) HDAC5-WT and -3SA vectors as templates (11). HDAC5 expression vectors used the cytomegalovirus promoter. *Mef2a* and *Mef2d* mRNA levels were reduced using the previously described AAV2 short hairpin RNA vectors (34). *Scn4b* mRNA levels were reduced using an AAV2 short hairpin RNA under the control of a U6 promoter. Control viruses included AAV2-GFP and AAV2-shLuciferase. All viruses were packaged by the University of South Carolina viral vector core and had titers >1 × 10^11^ viral genomes/μL.

### Stereotaxic Surgery

Stereotaxic surgery was performed as previously described (9). Coordinates to target the NAc were +1.6 mm anterior, +2.8 mm lateral, and −7.1 mm ventral from bregma (relative to skull) at a 10° angle. Viral placements were confirmed by anti-FLAG immunohistochemistry (IHC) (AAV-HDAC5), anti-mCherry IHC for AAV-shMef2d, and anti-GFP (green fluorescent protein) signal for AAV-shMef2a, AAV-shScn4b, and AAV-shLuciferase.

### Rat Intravenous Cocaine and Sucrose Self-Administration

All self-administration experiments were conducted as described previously (9), with the following modifications: During daily 2-hour (cocaine) or 1-hour (sucrose) sessions, rats were trained to press the active lever to receive a cocaine infusion (0.05 mL/infusion, 4 mg/mL cocaine) or a sucrose pellet. Rats were run on an escalating fixed ratio (FR) schedule (i.e., FR1, FR3, and FR5). After 1 week of home-cage abstinence and extinction training to criteria, rats were subjected to a cue test and a prime reinstatement test (cocaine). Viral placements were confirmed by a blinded experimenter, and rats with inaccurate viral injections or with insufficient viral expression were excluded prior to data analysis.

### Cell Culture

Human embryonic kidney cells (HEK293T) were cultured as previously described (9). HEK cells were transfected using the calcium phosphate method 24 hours after plating and allowed to incubate for 48 hours. Embryonic striatal neuron (E18/E19) cultures were taken from Long Evans rats and cultured as previously described (11). Cultured striatal neurons were transfected using the calcium phosphate method on day 7 in vitro and allowed to incubate for 48 hours.

### 3XFLAG-HDAC Immunoprecipitation

**Figure 1.**
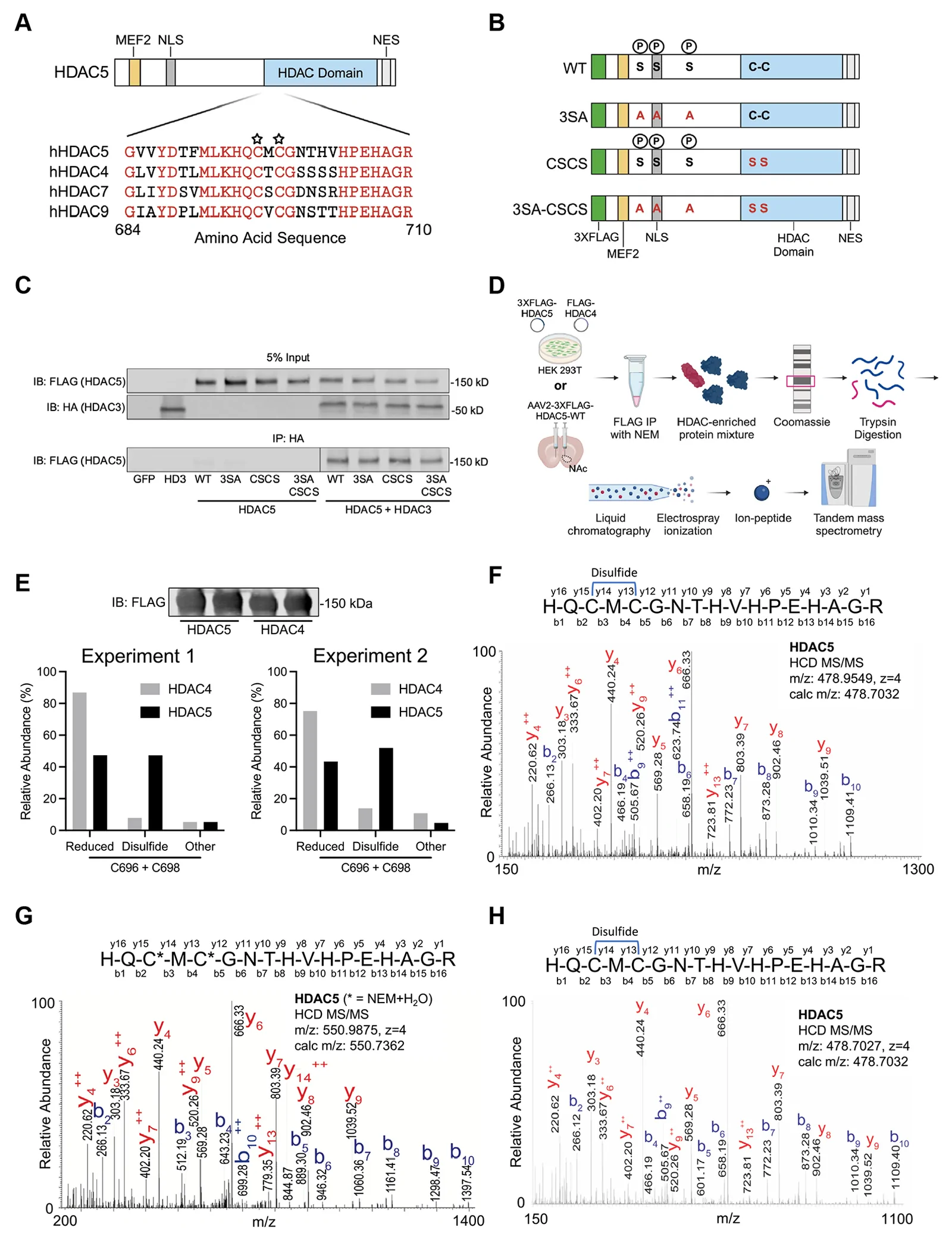
HDAC5 C696/C698 cysteines are not required for HDAC5-HDAC3 association and form an intramolecular disulfide bond. **(A)** Top: Domain structure of HDAC5 depicting MEF2 interaction domain (MEF2, yellow), NLS (dark gray), HDAC enzymatic domain (blue), and NES (light gray). Bottom: Amino acid sequence of class IIa HDACs aligned with amino acids 684 to 710 of HDAC5. Conserved amino acids are shown in red. Within the HDAC enzymatic domain, class IIa HDACs harbor conserved cysteines in the motif CXC (stars). **(B)** AAV-HDAC5 domain diagram. N-terminal 3XFLAG-tagged HDAC5 (green) AAV expression constructs were cloned. Mutations are shown in red. HDAC5-WT retains serine phosphorylation sites S259, S279, S498 (S, serine; encircled P, phosphate) and disulfide bonded cysteines C696 and C698 (C). HDAC5-3SA (3SA) harbors S259A/S279A/S498A mutations, HDAC5-CSCS (CSCS) harbors C696S/C698S mutations, and HDAC5-3SA-CSCS (3SA-CSCS) harbors S259A/S279A/S498A and C696S/C698S mutations. **(C)** Immunoblot of HDAC5-HDAC3 co-immunoprecipitation. **(D)** Schematic of targeted LC-MS/MS workflow. 3XFLAG-HDAC5 or FLAG-HDAC4 were transfected into HEK-293T cells or virally expressed in rat NAc before immunoprecipitation in NEM-containing buffer. Alkylated proteins were then analyzed using LC-MS/MS. **(E)** Top: Immunoblot of HDAC5 and HDAC4 expressed in HEK-293T cells. Bottom: Quantification of MS1 spectra of the CXC motif-containing tryptic fragment of HDAC5 and HDAC4 overexpressed in HEK-293T cells and immunoprecipitated followed by high-pressure LC-MS/MS in 2 separate experiments. **(F)** HEK-293T cell-derived HDAC5 MS2 spectrum consistent with an intramolecular disulfide bond between C696 and C698. **(G)** HEK-293T cell-derived HDAC5 MS2 spectrum consistent with NEM-alkylated (reduced) CXC cysteines. **(H)** HDAC5 MS2 spectrum consistent with disulfide bonded CXC cysteines from AAV2-HDAC5 expression in rat NAc. AAV, adeno-associated virus; GFP, green fluorescent protein; HA, hemagglutinin; HCD, higher-energy collisional dissociation; HDAC, histone deacetylase; HEK, human embryonic kidney; IB, immunoblot; IP, immunoprecipitation; LC-MS/MS, liquid chromatography–tandem mass spectrometry; MEF2, myocyte enhancer factor 2; NAc, nucleus accumbens; NES, nuclear export sequence; NLS, nuclear localization sequence; WT, wild-type.

Cells or NAc tissue were placed in lysis buffer, sonicated on ice, and centrifuged (16,000 RCF) at 4 °C. The supernatant was incubated with anti-FLAG M2 agarose beads (Millipore F2426) with rotation at 4 °C. The protein-conjugated beads were then washed with lysis buffer and incubated with 2XSAB at 65 °C to elute proteins. For the mass spectrometry (MS) experiments, the lysis buffer contained 40 mM n-ethylmaleimide (Sigma) to alkylate free cysteines. Following sonication, the homogenate was incubated for 1 hour at room temperature (RT) to alkylate. In experiment 1 (Figure 1E), the protein-conjugated beads were incubated with 2XSAB containing 50 mM TCEP for 15 minutes at 65 °C and 45 minutes at RT before incubating with 10 mM iodoacetamide (IAA, Thermo Fisher Scientific) for 30 minutes at RT. The TCEP and IAA alkylation steps were removed for experiment 2 (Figure 1E).

### Liquid Chromatography–MS/MS of HDAC4 and HDAC5

Immunoprecipitated HDAC was analyzed using an Easy-nLC 1200 liquid chromatography (LC) system coupled to an Orbitrap Fusion Lumos MS (Thermo Fisher Scientific) as described previously (35). To probe relative modification of the CXC cysteines, the signal intensities of the modified peptides (reduced vs. disulfide linked) were compared with the total signal for all forms of the CXC peptides observed to yield an estimate of percentage modification. For HDAC5 protein expressed in rat NAc, it was immunoprecipitated and prepared as described above. An internal standard–triggered parallel reaction monitoring method was developed using the SureQuant acquisition mode on the Orbitrap Fusion Lumos MS (Thermo Fisher Scientific). Heavy, stable isotope-encoded peptides of the HDAC5 CMC motif with and without oxidized methionine at position 4 (HQCMCGNTHVHPEHAGR*, R* = arginine [^13^C_6_, ^15^N_4_]) were synthesized and purified by high-pressure LC (95%) (LubioScience GmbH) and analyzed by LC-MS/MS in survey mode. C-terminal y-ion fragments (y^4^-y^8^) and an N-terminal (b^8^) fragment ion were used to build the SureQuant acquisition method (Supplement). Synthetic peptides were spiked into trypsin-digested HDAC5 isolated from NAc prior to LC–MS/MS analysis.

### Molecular Dynamics Simulations

Simulations were performed using AMBER20 with the ff14SB force field with additional acetyl-lysine parameters (36,37). Zn^21^ ions were handled using the parameters from the Li and Merz LJ12-6-4 potentials for divalent ions (38). The initial structure of the HDAC5 catalytic domain (residues 680–1083) was obtained from the AlphaFold database (39). All systems were simulated in 4 replicates of 260 ns.

### Deacetylase Activity Assay

HEK293T cells were transfected with 3XFLAG-HDAC5 constructs, which were then immunoprecipitated as described above. The HDAC5-conjugated beads were then equilibrated with the class IIa HDAC deacetylase activity kit (BPS Biosciences #50065) assay buffer, and successive volumes were loaded onto a black, low-binding NUNC microtiter plate. The kit was then used as directed, and fluorescence was measured using a SpectraMax M5 plate reader (Molecular Devices).

### Western Blotting

Samples were solubilized in 2XSAB containing 10% beta mercaptoethanol and incubated at 65 °C for 15 minutes. Western blotting was performed as previously described (9). Blots were imaged on LI-COR Odyssey CLx and analyzed with ImageStudio software.

### Immunohistochemistry

IHC was performed as previously described (9). Tissue was imaged on a Leica Thunder 3-dimensional (3D) Tissue Imager. Images were processed with ImageJ.

### Nuclear/Cytoplasmic Ratio

AAV2-HDAC5 mutants were expressed in rat NAc for 3 weeks, and anti-FLAG IHC was conducted as described above. All fluorescent cells in a 203 field were scored as nuclear, cytoplasmic, or both under blinded conditions.

### Electrophysiology

Rats were live decapitated. The brain was removed rapidly from the skull, and acute coronal slices (300-mm thickness) containing NAc were obtained using slicer Leica VT1200s. Slices were prepared as previously described (9). Virally infected cells were identified by red or green fluorescence. NAc MSNs were distinguished from interneurons by morphology and confirmed by membrane potential (up to −65 to −85 mV) and spiking patterns. In whole-cell voltage-clamp mode, action potentials (APs) were initiated by a series of depolarizing current pulses of increasing intensities (0–500 pA in 20 pA steps; 1-second duration at 0.2 Hz) and recorded with a holding transmembrane current clamped at −70 mV.

### Statistics

All statistics were performed using GraphPad Prism. All statistical tests are reported in Statistics Table.

## RESULTS

### HDAC5 Enzyme-Domain Cysteines Are Required for Intrinsic Deacetylase Activity

With the intention of testing whether HDAC5’s association with HDAC3 is required for its ability to limit cued cocaine seeking, we mutated 2 conserved cysteines found in the deacetylase domain (C696S/C698S or “CSCS”) (Figure 1A, B) that were predicted to disrupt HDAC3 binding (40,41). However, the CSCS mutations in wild-type HDAC5 or a nuclear-enriched HDAC5 mutant (i.e., S259A/S279A/S498A or “3SA”) (Figure S1A, B) failed to alter HDAC5:HDAC3 binding (Figure 1C). These 2 cysteines, which reside in a reduced state in HDAC4, help coordinate a structural zinc ion in the deacetylase catalytic domain (40). Tandem mass spectrometry (Figure 1D) confirmed that the HDAC4 conserved cysteines were mostly reduced (~75%–85%), with ~10% of the peptides forming a disulfide bond (Figure 1E; Figure S1C, D; Tables S1–S4). Surprisingly, HDAC5 C696 and C698 formed an intramolecular disulfide bond in nearly half of the peptides (Figure 1E–G). HDAC4 and HDAC5 were immunoprecipitated at similar amounts (Figure 1E, top), and total protein-level analyses revealed similar combined peptide ion intensities, sequence coverage, and number of unique peptides (Table S5), supporting the noted difference in relative disulfide bond abundance. Using SureQuant internal standard, parallel reaction monitoring with heavy-labeled peptide standards (Figure S1E, F), we confirmed the presence of the HDAC5 disulfide-bonded cysteines in epitope-tagged HDAC5 expressed in rat NAc neurons in vivo (Figure 1H; Supplement).

**Figure 2.**
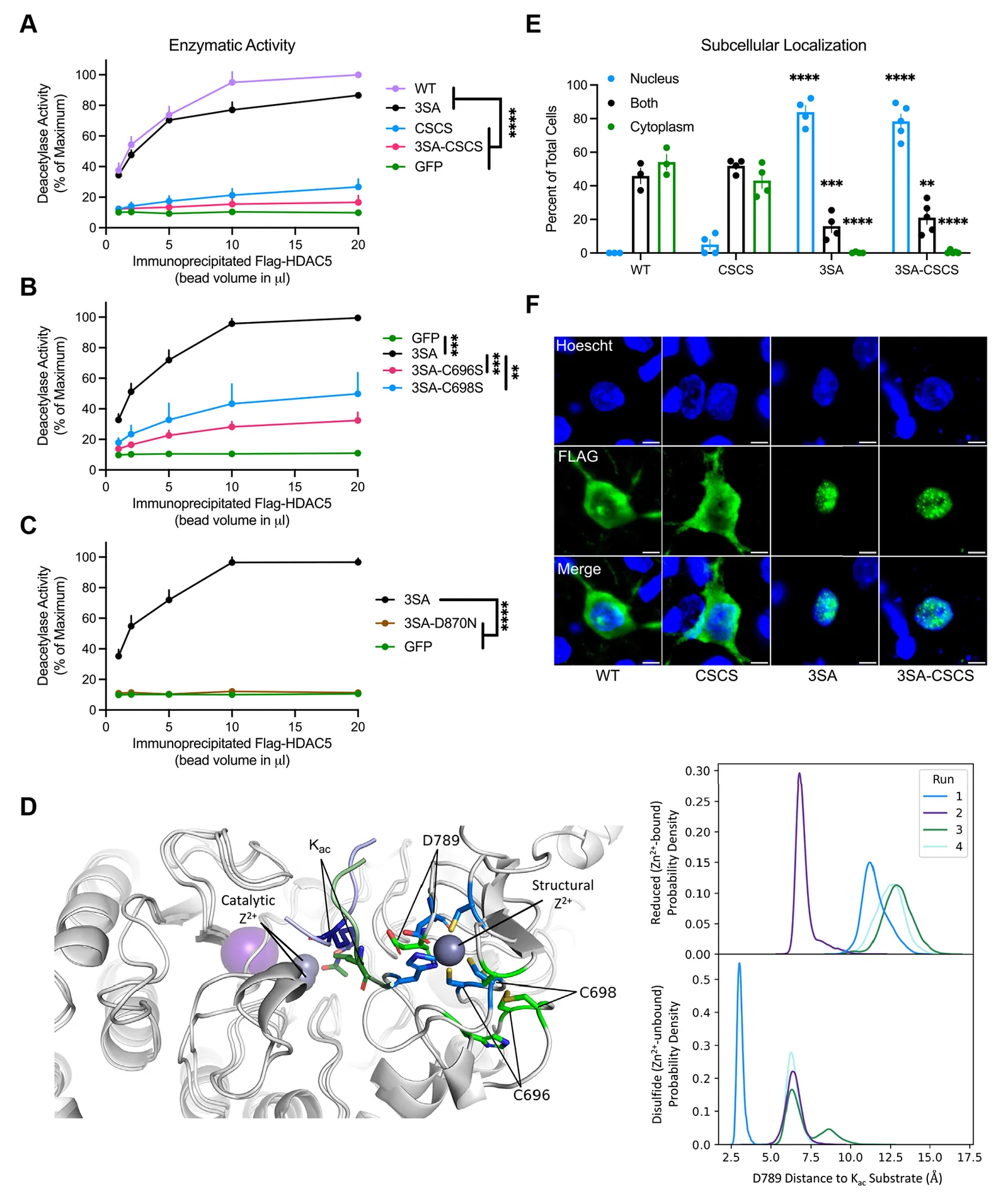
HDAC5 disulfide-bonded cysteines are required for intrinsic deacetylase activity but do not regulate subcellular localization. **(A)** HDAC5 enzymatic activity assay of CSCS and 3SA-CSCS mutants compared with GFP, WT, and 3SA controls (all groups: *n* = 4; mixed effects model [residual maximum likelihood] with Tukey’s post hoc test; ****p < .0001 WT vs. GFP, CSCS, 3SA-CSCS separately, 3SA vs. GFP, CSCS, 3SA-CSCS separately). **(B)** Enzymatic activity of single-cysteine mutants (3SA-C696S, 3SA-C698S) compared with HDAC5-3SA and GFP controls (all groups: *n* = 3; 2-way RM ANOVA with Tukey’s post hoc test; **p < .01 3SA vs. 3SA-C698S, ***p < .001 3SA vs. GFP, 3SA vs. 3SA-C696S). **(C)** Enzymatic activity of catalytic site mutant (3SA-D870N) compared with HDAC5-3SA and GFP controls (all groups: *n* = 3; 2-way RM ANOVA with Tukey’s post hoc test; ****p < .0001 3SA vs. 3SA-D870N, GFP separately). **(D)** Left: Model of average HDAC5 structures following molecular dynamics simulation with disulfide-bonded C696 and C698 (green residues) and reduced, zinc-coordinating C696 and C698 (blue residues) superimposed (K_ac_: H3K9ac peptide; gray sphere: Zn^2+^; purple sphere: K^+^). Right: Probability density of the distance between D789 side-chain oxygens and the H3K9ac nitrogen from 4 independent molecular dynamics simulations of reduced (top) and disulfide-bonded (bottom) HDAC5. **(E)** Quantification of HDAC5 mutant subcellular localization in rat NAc. All HDAC5-expressing cells in a 20× field were labeled as expressing HDAC5 in nuclear, cytoplasmic, or both subcompartments. Data is expressed as the percentage of total cells scored per rat (WT: *n* = 3 rats, CSCS: *n* = 4, 3SA: *n* = 4, 3SA-CSCS *n* = 5; 2-way ANOVA with Tukey’s post hoc test; **p < .01, ***p < .001, ****p < .0001 vs. HDAC5-WT). **(F)** Representative images of HDAC5 mutant subcellular localization in rat NAc. Hoescht staining delineated nuclei, and α-FLAG immunostaining labeled HDAC5 expression (scale bars = 5 μm). Data shown are mean ± SEM **(A–C, E)**; each point represents 1 rat **(E)**. ANOVA, analysis of variance; GFP, green fluorescent protein; HDAC, histone deacetylase; NAc, nucleus accumbens; RM, repeated-measures; WT, wild-type.

Given the role of the CXC cysteines in the class IIa HDAC-specific structural zinc coordination site and their proximity to the HDAC4 and HDAC7 catalytic pocket (40,42), we tested their role in HDAC5-associated deacetylase activity in vitro using a class IIa HDAC-optimized substrate. The cysteine mutants exhibited almost no deacetylase activity (Figure 2A and Figure S2A, B). Furthermore, single cysteine mutants (i.e., C696S and C698S) showed partial reductions (Figure 2B and Figure S2C, D), indicating that both cysteines are important for HDAC5 enzymatic activity. A catalytic site (40,42–44) mutant (HDAC5–3SA-D870N) also lacked deacetylase activity (Figure 2C and Figure S2E, F), suggesting that the lack of activity in the CSCS mutants was not due to disruption of co-immunoprecipitated proteins. We explored the potential enzymatic importance of the C696-C698 disulfide bond by performing molecular dynamics simulations of HDAC5 with and without disulfide-bonded cysteines using AlphaFold and the crystal structure of HDAC4 (39,40) (Protein Data Bank ID: 5A2S) and acetyl-lysine H3K9 (sequence: TARK_ac_ST) as a substrate (Figure 2D and Figure S2G, H). Aspartic acid D789 has been implicated in positioning substrate into the catalytic pocket and is required for HDAC4 deacetylase activity (40,43). We found that when C696 and C698 were in the reduced, zinc-bound state, D789 was turned away from the catalytic pocket and toward the structural zinc ion (Figure 2D and Figure S2G). Modeling a disulfide bond between C696 and C698 resulted in D789 turning toward the catalytic pocket, increasing the proximity of D789 to the acetyl-lysine H3K9 substrate (Figure 2D and Figure S2H), which may assist catalytic activity by facilitating substrate binding.

### HDAC5 Intrinsic Deacetylase Activity Is Required to Suppress Cue-Reinstated Cocaine Seeking

**Figure 3.**
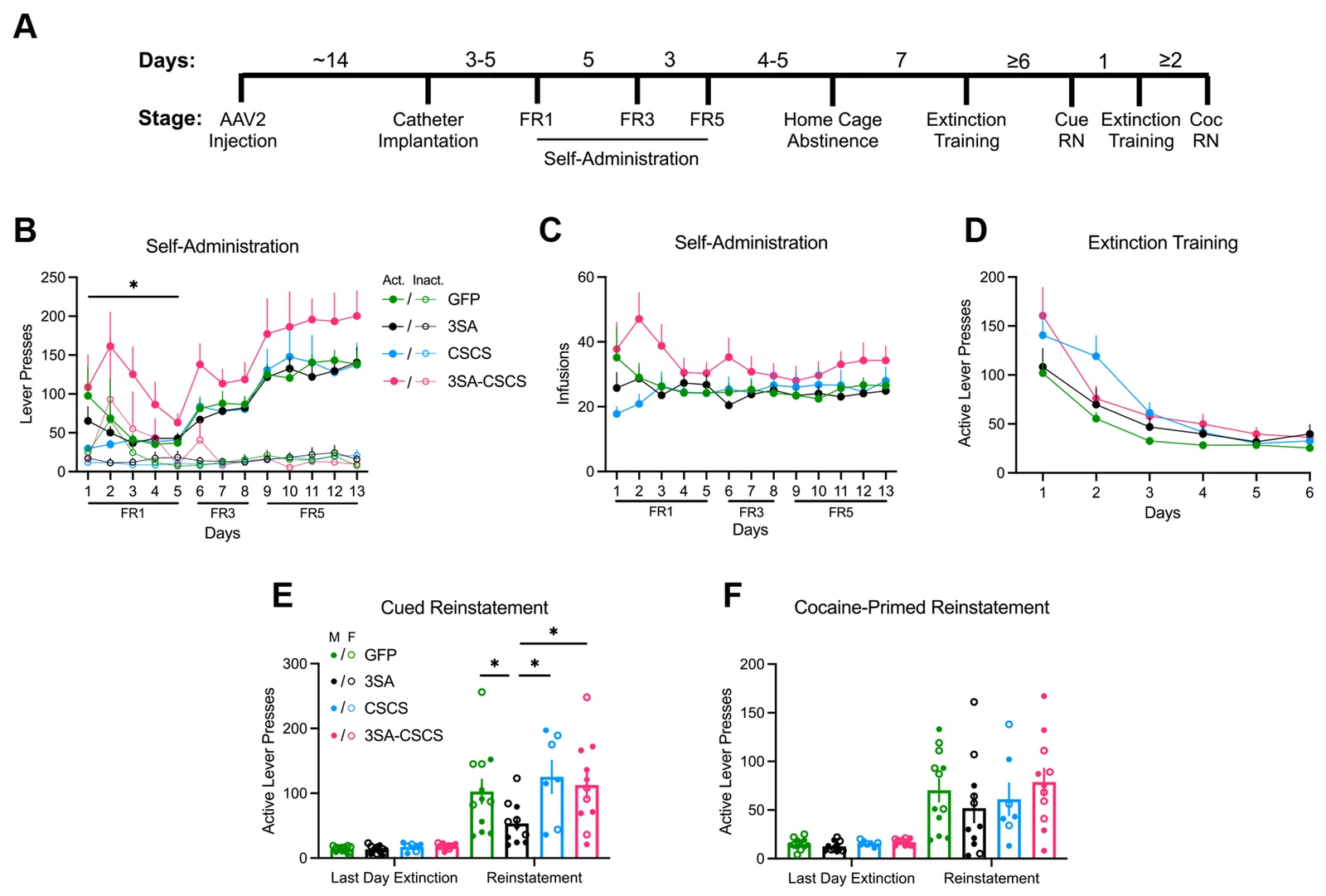
HDAC5 intrinsic enzymatic activity is required to limit cued reinstatement of cocaine seeking. **(A)** Experimental timeline. The number of days between each experimental stage is shown above the chart. **(B)** Active (filled circles) and inactive (open circles) lever presses during the cocaine self-administration stages of the timeline. The rats that received AAV2-HDAC5-3SA-CSCS showed higher active lever pressing during the FR1 stage before exhibiting no statistical differences in FR3 or FR5 (GFP: *n* = 12 [5 female, 7 male], 3SA: *n* = 12 [5 female, 7 male], CSCS: *n* = 8 [4 female, 4 male], 3SA-CSCS *n* = 12 [5 female, 7 male]; 2-way RM ANOVA with Tukey’s post hoc test; *p < .05 3SA-CSCS vs. CSCS, 3SA). **(C)** Cocaine infusions during self-administration (2-way RM ANOVA; no differences between groups observed). **(D)** Active lever presses during first 6 days of extinction. There was a significant interaction of virus and session, though no significant post hoc comparisons were observed (2-way RM ANOVA with Tukey’s post hoc test). **(E)** Active lever presses on the last day of extinction and cue-induced reinstatement of cocaine seeking (1-way ANOVA with Fisher’s least significant difference post hoc test; *p < .05 3SA vs. GFP, CSCS, 3SA-CSCS). **(F)** Active lever presses on the last day of the second stage of extinction and cocaine-primed reinstatement (10 mg/kg intraperitoneal). (GFP: *n* = 12 [5 female, 7 male], 3SA: *n* = 11 [5 female, 6 male], CSCS: *n* = 7 [3 female, 4 female], 3SA-CSCS: *n* = 11 [5 female, 6 male]; 1-way ANOVA; no differences between groups observed). Data shown are mean ± SEM **(B–F);** each point represents 1 rat **(E, F);** closed squares: male, open circles: female **(E, F)**. ANOVA, analysis of variance; FR, fixed ratio; GFP, green fluorescent protein; HDAC, histone deacetylase; RM, repeated-measures; RN, reinstatement.

Importantly, the presence of the CSCS mutations in either HDAC5-WT or -3SA did not alter the phosphorylation-dependent subcellular distribution of 3XFlag-HDAC5 in rat NAc neurons (Figure 2E, F and Figure S2I). Next, we tested whether the CSCS mutants altered HDAC5’s suppression of cue-induced cocaine seeking. We used AAV2-mediated NAc expression of HDAC5-3SA, HDAC5-CSCS, HDAC5-3SA-CSCS, or GFP in male and female rats and allowed them to self-administer cocaine (0.2 mg/infusion) on an escalating FR schedule (Figure 3A). Although the study was not powered to detect sex differences, the behavioral measures exhibited similar trends in males and females (Figure S3A–F). During FR1, the 3SA-CSCS group showed higher active-lever pressing than the 3SA and CSCS groups (Figure 3B), but this effect normalized during FR3 and FR5 (Figure 3B), and at no point in the study were there any differences in cocaine infusions (Figure 3C). Following 7 days of home-cage abstinence, no differences in extinction behavior were observed (Figure 3D). However, while HDAC5-3SA reduced cue-induced reinstatement of cocaine seeking, neither HDAC5-CSCS nor HDAC5-3SA-CSCS mutants affected cued seeking (Figure 3E), suggesting that HDAC5’s intrinsic deacetylase activity in NAc is required to limit cue-reinstated cocaine seeking.

### HDAC5 Limits Cue-Reinstated Cocaine Seeking Through an MEF2-Independent Mechanism in the NAc

**Figure 4.**
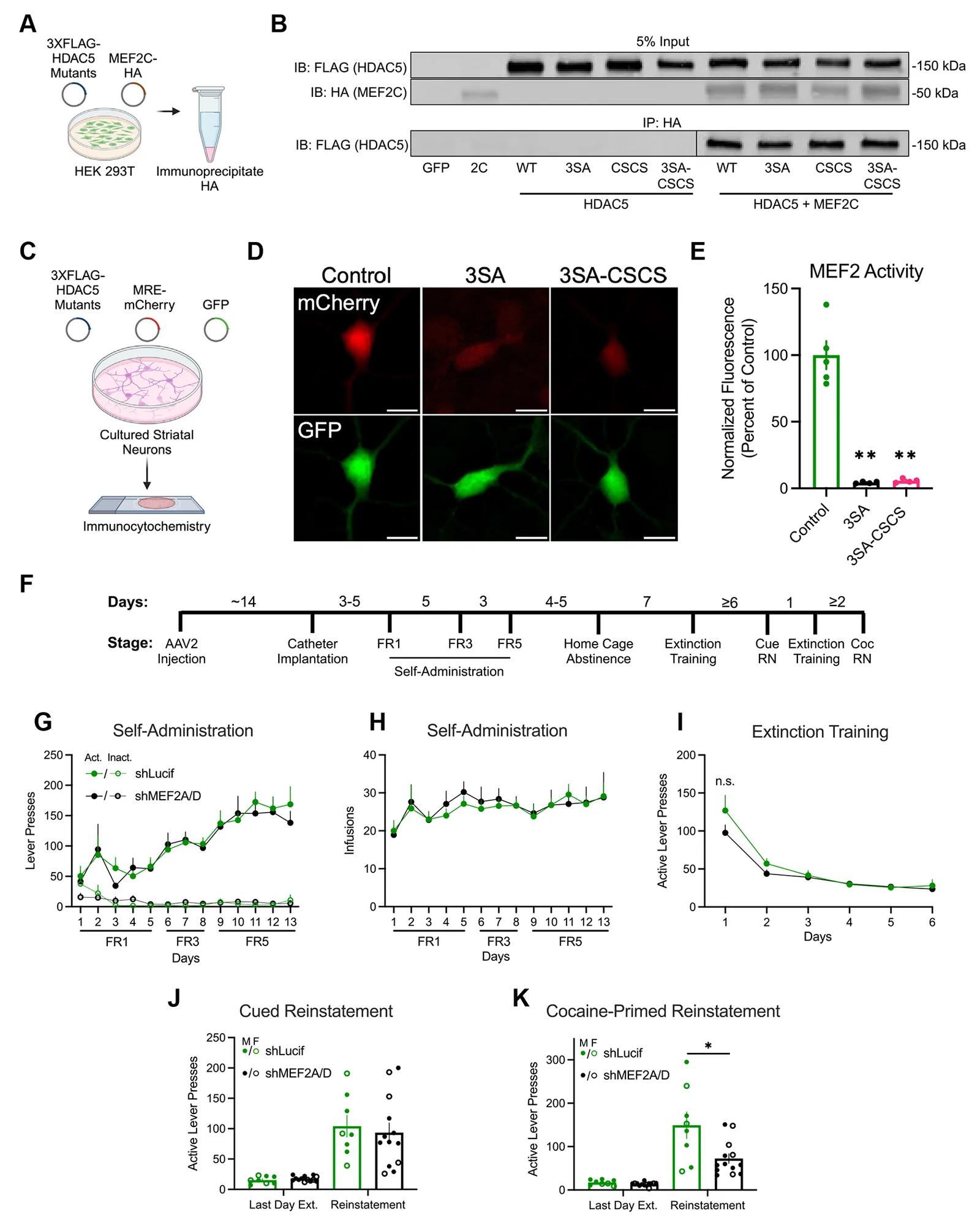
HDAC5 limits cued cocaine seeking through an MEF2-independent mechanism in the NAc. **(A)** Schematic of experimental procedure done in **(B)**. **(B)** Immunoblot of HDAC5-MEF2C co-immunoprecipitation. **(C)** Schematic of experimental procedure done in **(D)** and **(E)**. **(D)** Representative images of cultured striatal neurons expressing mCherry under the control of a MEF2 response element, GFP, and HDAC5 mutant constructs. **(E)** Quantification of MEF2 transcriptional activity measured by mCherry fluorescence normalized to GFP and expressed as percentage of transfection control (control: *n* = 5, 3SA, CSCS, 3SA-CSCS: *n* = 4; Brown–Forsythe and Welch’s ANOVA tests with Dunnet’s T3 post hoc test; *p < .05, **p < .01 vs. control). **(F)** Experimental timeline. Rat intravenous cocaine self-administration following short hairpin RNA-mediated reduction of MEF2A and MEF2D in the NAc. **(G)** Active (filled circles) and inactive (open circles) lever presses by day during the self-administration stages of the experiment (shLucif: *n* = 9 [3 female, 6 male], shMEF2A/D: *n* = 13 [4 female, 9 male]; 2-way RM ANOVA; no differences between groups observed). **(H)** Cocaine infusions by day during the self-administration stages of the experiment (2-way RM ANOVA; no differences between groups observed). **(I)** Active lever presses by day during extinction (2-way RM ANOVA; no differences between groups observed; n.s.). **(J)** Active lever presses on the last day of extinction and during cue-induced reinstatement of cocaine seeking (shLucif: *n* = 8 [5 male, 3 female], shMEF2A/D: *n* = 13 [9 male, 4 female]; unpaired 2-tailed *t* test; no differences between groups observed). **(K)** Active lever presses on the last day of the second stage of extinction and cocaine-primed reinstatement (unpaired 2-tailed *t* test; *p < .05). Data shown are mean ± SEM **(E, G–K);** each point represents 1 cell-culture well **(E)** or 1 rat **(J, K);** closed circles: male, open circles: female **(J, K)**. AAV, adeno-associated virus; ANOVA, analysis of variance; FR, fixed ratio; GFP, green fluorescent protein; HA, hemagglutinin; HDAC, histone deacetylase; HEK, human embryonic kidney; IB, immunoblot; IP, immunoprecipitation; NAc, nucleus accumbens; n.s., not significant; RM, repeated-measures; RN, reinstatement; WT, wild-type.

Class IIa HDACs associate with genomic DNA indirectly by binding to transcription factors, including the MEF2 family, and MEF2 proteins in the NAc can modulate noncontingent cocaine behavior (34,45–47). We observed that the CSCS mutants did not alter HDAC5-MEF2C binding (Figure 4A, B)or HDAC5’s ability to reduce MEF2-dependent gene expression in cultured primary striatal neurons (Figure 4C–E), which has been reported to be independent of class IIa HDAC deacetylase domain (34,48,49). Consistent with these findings, we observed that viral-mediated knockdown of NAc MEF2A and MEF2D (Figure S4A–C) had no effects on acquisition of cocaine self-administration (Figure 4F–H), extinction training (Figure 4I), or cue-reinstated cocaine seeking (Figure 4J). However, MEF2A/MEF2D knockdown did significantly reduce cocaine prime-reinstated seeking (Figure 4K). Finally, there were no apparent sex differences when the data were disaggregated (Figure S4D–I). Together, these data suggest that HDAC5 intrinsic deacetylase activity limits cue-reinstated cocaine seeking through a MEF2-independent mechanism in the rat NAc, but that cocaine-primed seeking is dependent, at least in part, on NAc MEF2A and MEF2D.

### HDAC5 Intrinsic Deacetylase Activity Limits NAc MSN Excitability Through Repression of *Scn4b*

**Figure 5.**
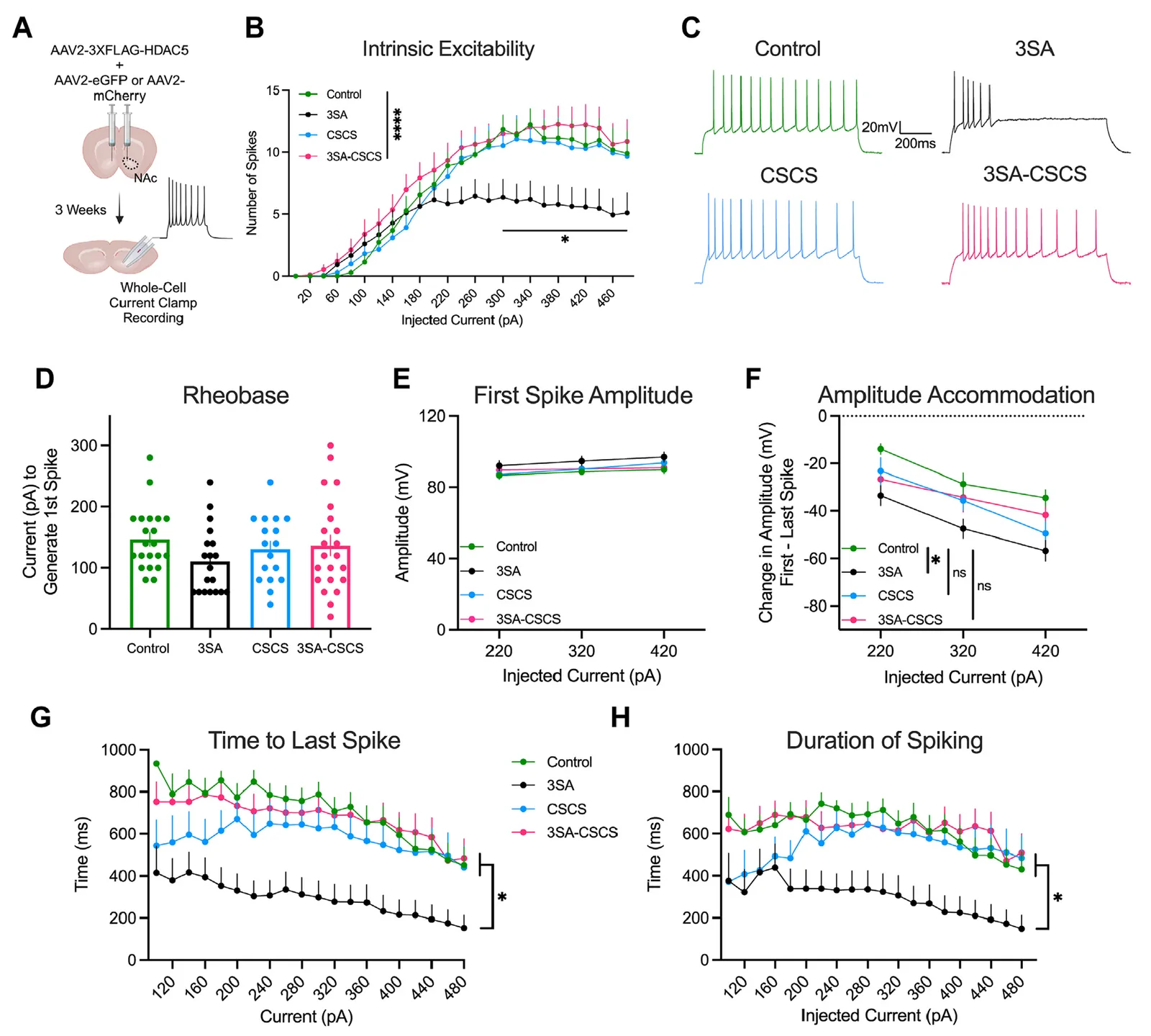
HDAC5 intrinsic enzymatic activity limits MSN intrinsic excitability. **(A)** Schematic of experimental procedure in **(B–H)**. Control: *n* = 22 cells/4 rats, 3SA: *n* = 19 cells/4 rats, CSCS: *n* = 17 cells/4 rats, 3SA-CSCS: *n* = 22 cells/4 rats for all experiments. **(B)** Intrinsic excitability of NAc MSNs at increasing current injections (2-way RM ANOVA with Tukey’s post hoc test; ****p < .0001 injected current × virus interaction, *p < .05 3SA vs. control pairwise comparisons). **(C)** Representative traces at 300-pA current injection. **(D)** Rheobase defined as the lowest current step to elicit a spike (1-way ANOVA; no differences between groups observed). **(E)** Amplitude of the first spike elicited at 220, 320, and 420-pA current injections (mixed effects model [REML]; no differences between groups observed). **(F)** Change in amplitude between the first and last spike elicited at 220, 320, and 420-pA current injections (mixed effects model [REML] with Tukey’s post hoc test; *p < .05 3SA vs. control). **(G)** Time from start of 1000-ms current injection to the peak of the last spike (mixed effects model [REML] with Tukey’s post hoc test; *p < .05 3SA vs. control, CSCS, and 3SA-CSCS separately). **(H)** Time of spike train from peak of first spike to peak of last spike during 1000-ms current injection (mixed effects model [REML] with Tukey’s post hoc test; *p < .05 3SA vs. control, CSCS, and 3SA-CSCS separately). Data shown are mean ± SEM **(B, D–H)**; each point represents 1 rat **(D)**. ANOVA, analysis of variance; HDAC, histone deacetylase; MSN, medium spiny neuron; NAc, nucleus accumbens; ns, not significant; REML, residual maximum likelihood; RM, repeated-measures.

HDAC5 suppresses NAc MSN firing rate without altering the rheobase (9), but the relevance of this effect to cue-reinstated drug seeking remains unclear. Using viral-mediated NAc expression of HDAC5-3SA, -CSCS, -3SA-CSCS, or GFP in rats (Figure 5A), we replicated our finding that HDAC5-3SA significantly reduces the NAc MSN firing rate (Figure 5B, C). However, the cysteine mutants failed to alter NAc MSN excitability (Figure 5B–D). No changes in rheobase were observed in any of the groups (Figure 5D). Further analysis of the spike trains at 3 representative current injections (i.e., 220, 320, and 420 pA) revealed no significant differences in the first-spike amplitude (Figure 5E). However, HDAC5-3SA, but not HDAC5-CSCS or HDAC5-3SA-CSCS, produced a decrease in amplitude between the first and last spike (Figure 5F) and in the first 3 spikes (Figure S5A–C), the time to last spike (Figure 5G), and the duration of spiking (Figure 5H). Thus, HDAC5 limited NAc MSN excitability in a deacetylase-dependent manner, likely through a mechanism that involves amplitude accommodation and depolarization block.

**Figure 6.**
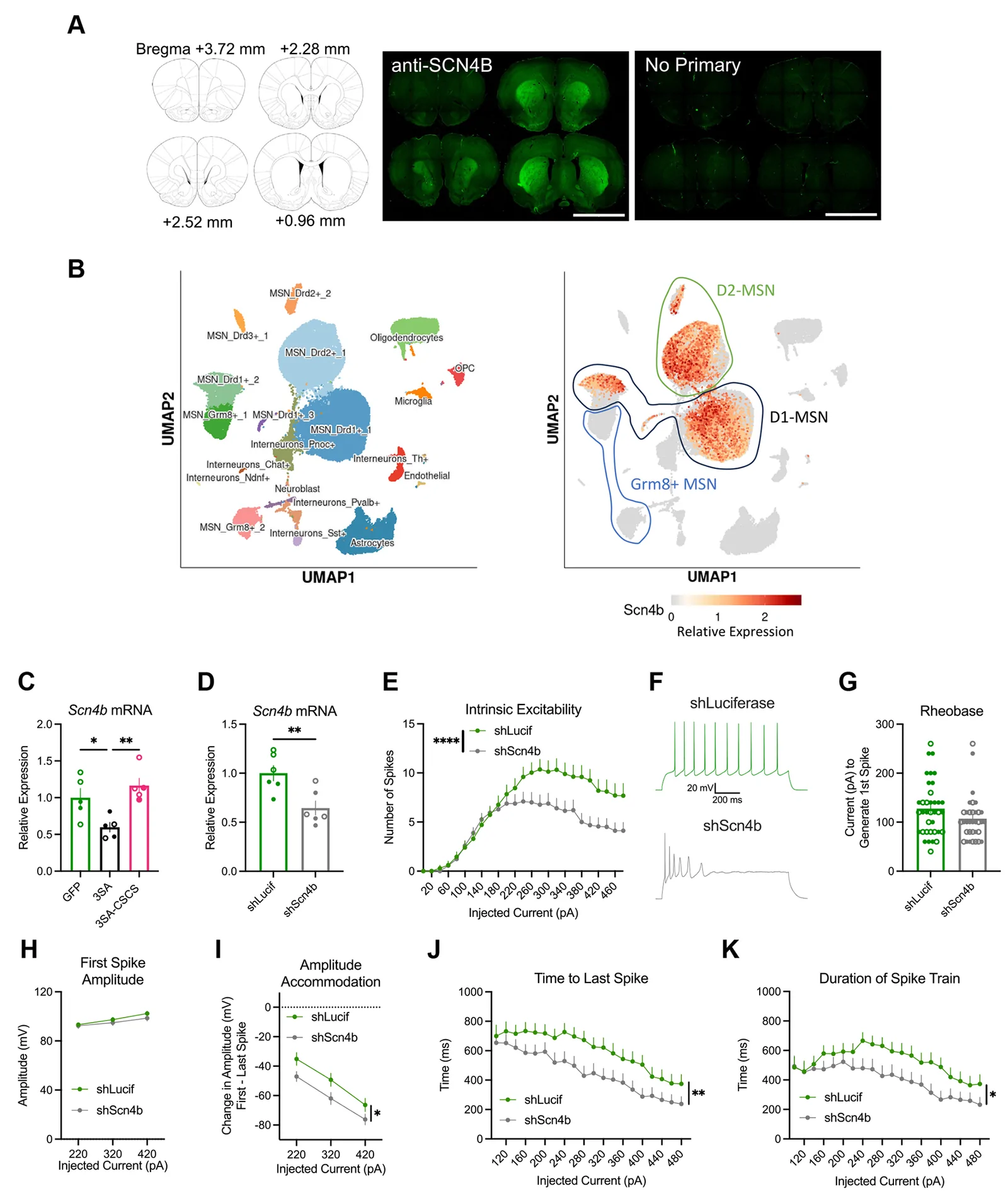
Reduction of HDAC5 target gene *Scn4b* limits MSN excitability. **(A)** SCN4B expression in rat forebrain, coronal slices (scale bar = 5000 μm). **(B)** UMAP plot of single-nucleus RNA sequencing of NAc with cell types labeled (left) and heatmap of Scn4b relative expression (right; D1-MSNs, D2-MSNs, Grm81 MSNs). Data obtained from Hughes *et al.*, 2024 (50). **(C)** *Scn4b* mRNA levels in rat NAc following HDAC5 expression (GFP: *n* = 5 [2 male, 3 female], 3SA *n* = 5 [3 male, 2 female], 3SA-CSCS: *n* = 5 [2 male, 3 female]; 1-way ANOVA with Tukey’s post hoc test; *p < .05 GFP vs. 3SA, **p < .01 3SA vs. 3SA-CSCS). **(D)** *Scn4b* mRNA levels in rat NAc following shRNA expression (shLucif: *n* = 6 [3 female, 3 male], shScn4b *n* = 6 [3 female, 3 male]; unpaired 2-tailed *t* test; **p < .01). **(E)** Intrinsic excitability of NAc MSNs following shRNA expression (2-way RM ANOVA with Tukey’s post hoc test; ****p < .0001 injected current × virus interaction). **(F)** Representative traces at 300-pA current injection. **(G)** Rheobase defined as the lowest current step to elicit a spike (unpaired 2-tailed *t* test; no differences between groups observed). **(H)** Amplitude of the first spike elicited at 220, 320, and 420-pA current injections (mixed effects model [REML]; no differences between groups observed). **(I)** Change in amplitude between the first and last spike elicited at 220, 320, and 420-pA current injections (2-way RM ANOVA; *p < .05 shScn4b vs. shLucif). **(J)** Time from start of 1000-ms current injection to peak of last spike (mixed effects model [REML]; **p < .01 shScn4b vs. shLucif). **(K)** Time of spike train from the peak of the first spike to the peak of the last spike during 1000-ms current injection (mixed effects model [REML]; *p < .05 shScn4b vs. shLucif). **(E–K)** shLucif: *n* = 37 cells (22 male, 15 female)/10 rats (5 male, 5 female), shScn4b: *n* = 34 cells (18 male, 16 female)/10 rats (5 male, 5 female). Data shown are mean ± SEM **(C–E, G–K)**; each point represents 1 rat **(C, D, G)**; closed squares: male, open circles: female **(C, D, G)**. ANOVA, analysis of variance; D1-MSN, D1-expressing MSN; D2-MSN, D2-expressing MSN; GFP, green fluorescent protein; HDAC, histone deacetylase; mRNA, messenger RNA; MSN, medium spiny neuron; NAc, nucleus accumbens; OPC, oligodendrocyte progenitor cell; REML, residual maximum likelihood; RM, repeated-measures; shRNA, short hairpin RNA; UMAP, uniform manifold approximation and projection.

Previous analysis of HDAC5-3SA–repressed transcripts in rat NAc MSNs revealed enrichment of genes linked to voltage-gated ion channels and intrinsic excitability (9), including *Scn4b*, which encodes an auxiliary subunit of voltage-gated sodium channels (24–26). IHC revealed strong SCN4B striatal enrichment (Figure 6A), and analysis of *Scn4b* mRNA expression in a single-nucleus RNA sequencing dataset from mouse NAc (50) showed restricted expression in D1-expressing and D2-expressing MSNs (Figure 6B). Furthermore, HDAC5-3SA, but not HDAC5-3SA-CSCS (Figure 6C) or HDAC5-CSCS (Figure S6A), significantly reduced *Scn4b* mRNA levels in the NAc, indicating that HDAC5’s deacetylase activity is required to reduce *Scn4b* mRNA expression. To test the influence of Scn4b on NAc MSN excitability, we designed and validated a viral-mediated short hairpin RNA to reduce *Scn4b* expression (AAV2-shScn4b) in rat NAc (Figure 6D and Figure S6B). Like HDAC5-3SA, AAV2-shScn4b significantly reduced MSN excitability, increased spike amplitude accommodation, reduced time to last spike, and reduced the duration of the spike train, but without altering rheobase or first-spike amplitude (Figure 6E–K and Figure S6H–J). Notably, no appreciable sex differences were observed when rats were disaggregated by sex (Figure S6C–J).

### NAc *Scn4b* Is Required for Cue-Induced Cocaine Seeking but Not Sucrose Seeking

**Figure 7.**
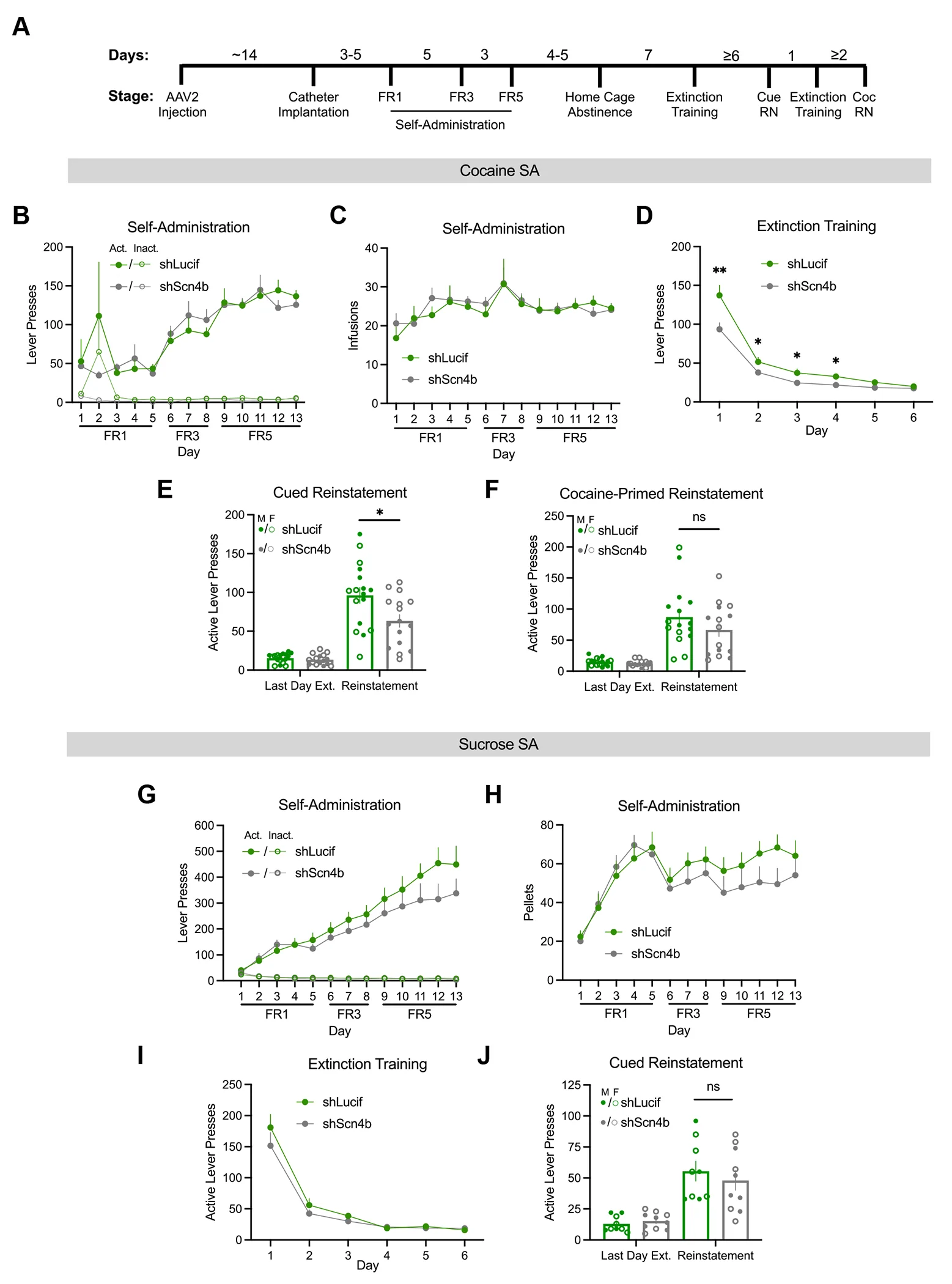
Reduction of HDAC5 target gene *Scn4b* limits cued reinstatement of cocaine, but not sucrose, seeking. **(A)** Experimental timeline of the data presented in **(B–F)**. **(B)** Lever presses during the cocaine self-administration stages of the experiment (closed circles: active lever, open circles: inactive lever; shLucif: *n* = 17 [9 males, 8 females], shScn4b *n* = 16 [7 males, 9 females]; FR1 and FR3 analyzed by 2-way RM ANOVA; FR3 analyzed by mixed effects model [REML]; no difference between groups observed). **(C)** Cocaine infusions by day during self-administration stages of experiment (mixed effects model [REML]; no difference between groups observed). **(D)** Active lever presses during extinction training (2-way RM ANOVA; *p < .05, **p < .01 shLucif vs. shScn4b pairwise comparisons). **(E)** Active lever presses during the last day of extinction and cue-induced reinstatement (unpaired 2-tailed *t* test; *p < .05). **(F)** Active lever presses during cocaine-primed reinstatement (unpaired 2-tailed *t* test; no difference between groups observed; ns). **(G)** Lever presses during the sucrose self-administration stages of the experiment (closed circles: active lever, open circles: inactive lever; shLucif: *n* = 9 [4 males, 5 females], shScn4b *n* =10 [5 males, 5 females]; FR1, FR3, FR5 analyzed by 2-way RM ANOVA; no difference between groups observed). **(H)** Sucrose pellets by day during self-administration stages of experiment (2-way RM ANOVA; no difference between groups observed). **(I)** Active lever presses during extinction training (2-way RM ANOVA; no difference between groups observed). **(J)** Active lever presses during the last day of extinction and cue-induced reinstatement (unpaired 2-tailed *t* test; no difference between groups observed; ns). Data shown are mean ± SEM **(B–J)**; each point represents 1 rat **(E, F, I, J)**; closed squares: male, open circles: female **(E, F, J)**. AAV, adeno-associated virus; ANOVA, analysis of variance; FR, fixed ratio; HDAC, histone deacetylase; ns, not significant; REML, residual maximum likelihood; RM, repeated-measures; RN, reinstatement; SA, self-administration.

To test whether NAc *Scn4b* is required for cue-reinstated cocaine seeking, we infused AAV2-shScn4b bilaterally into the NAc of adult rats and allowed them to self-administer cocaine (Figure 7A). We observed no significant effects on acquisition of cocaine self-administration compared with AAV2-shLuciferase controls (Figure 7B, C). However, shScn4b reduced both context-associated cocaine seeking during extinction training (Figure 7D) and cue-reinstated cocaine seeking (Figure 7E). We observed no effects of shScn4b on prime-reinstated cocaine seeking (Figure 7F), and although not powered for sex differences like all previous experiments in this study, behavioral measures appeared similar between the sexes (Figure S7A–F). Finally, because NAc HDAC5 limits cue-reinstated cocaine- or heroin-seeking, but not sucrose-seeking, behavior (9,11), we tested NAc shScn4b in sucrose self-administration and cue-reinstated sucrose seeking. We observed that knockdown of NAc Scn4b had no significant effects on any aspect of sucrose self-administration or seeking (Figure 7G–J and Figure S8A–F), suggesting that SCN4B participates in a drug-selective plasticity mechanism to support cue-reinstated seeking.

## DISCUSSION

We found here that 2 conserved cysteines, C696 and C698, located within HDAC5’s enzymatic domain form an intramolecular disulfide bond and are essential for HDAC5’s intrinsic deacetylase activity, but they do not impact binding to the HDAC3 co-repressor, phosphorylation-dependent nucleocytoplasmic shuttling, or repression of MEF2-dependent transcription. We showed that HDAC5’s deacetylase activity suppresses NAc MSN excitability, limits cue-reinstated cocaine seeking, and is required, directly or indirectly, to limit the expression of NAc *Scn4b*. Like HDAC5-3SA expression, reduction of NAc *Scn4b* is sufficient to reduce both MSN intrinsic excitability and cue-reinstated cocaine, but not sucrose, seeking. Therefore, our findings strongly suggest that the HDAC5/SCN4B-regulated NAc MSN firing rate supports a drug-selective plasticity mechanism that underlies prepotent drug-cue associations and relapse-like drug-seeking behavior.

Previous studies of class IIa HDACs revealed that they exhibit low intrinsic enzymatic activity on histones compared with class I HDAC binding partners (18–20). Based on these studies, we initially hypothesized that HDAC5 functions as a scaffold to recruit HDAC3, a class I HDAC, and that HDAC3’s enzymatic activity was critical for HDAC5’s behavioral effects. To our surprise, we found that while the HDAC5 CSCS mutants exhibited almost no deacetylase activity, they retained their ability to bind HDAC3, which led us to conclude that the mutation disrupts HDAC5’s intrinsic deacetylase activity. However, we cannot rule out the possibility that the CSCS mutation disrupts HDAC5 binding to some other factor that exhibits deacetylase activity (51,52) or that it confers a gain-of-function recruitment of an inhibitory protein. Furthermore, the relevant HDAC5 substrate(s) in NAc MSNs are not yet clear. It is possible that HDAC5 acts on a nonhistone substrate (53,54), such as a transcription factor or co-repressor, to reduce drug-seeking behavior.

In a previous study, it was found that cardiomyocyte HDAC4 formed a dynamic disulfide bond between the CXC cysteines following exposure to oxidative stress, which promotes HDAC4 nuclear export and transcriptional changes that support cardiac hypertrophy (55). We found that HDAC5 exists basally with a high proportion of disulfide-bonded species and, unlike HDAC4 (55), mutation of the HDAC5 cysteines had no detectable influence on its subcellular localization in rat NAc, revealing key differences between closely related class IIa HDACs or cell type–specific influences.

How the HDAC5 CXC cysteines and the disulfide bond between them influence deacetylase activity is unclear. Crystal structures of HDAC4 and HDAC7 (40,42) show that the cysteines in a reduced, unmodified form coordinate a structural zinc ion that is likely important for enzymatic activity, but a crystal structure does not exist for HDAC5. Our modeling of HDAC5’s 3D structure suggests that a disulfide bond between C696 and C698 may facilitate catalytic activity by increasing the proximity of acetylated substrate to amino acid D789—a critical residue thought to guide substrate to the catalytic pocket (43). Mutation of the individual cysteines reduced, but did not eliminate, HDAC5 deacetylase activity, suggesting that the cysteines influence deacetylase activity independent of the C696-C698 disulfide bond.

The MEF2 family of transcription factors are well-described targets of class IIa HDACs, which bind to MEF2 proteins via their N-terminal domain and repress MEF2 transcriptional activity (46,47). This repressor activity occurs independently of class IIa HDAC deacetylase activity (48,49). The predominant isoforms in the NAc, MEF2A and MEF2D (27,34), influence MSN dendritic spine density, cocaine conditioned place preference, and cocaine locomotor sensitization (34), but their roles in cocaine self-administration and drug seeking had not been explored. We found that NAc MEF2A and MEF2D were required for drug prime-reinstated, but not cue-reinstated, cocaine seeking. The lack of involvement of MEF2 in cue-reinstated cocaine seeking is consistent with our observations that HDAC5-3SA-CSCS still binds to MEF2C and represses MEF2-dependent transcription.

HDAC5 reduces intrinsic excitability and several ion-channel genes in both D1-and D2-class MSNs (9). The characteristic depolarization block and amplitude accommodation that HDAC5 produces in NAc MSNs, HDAC5’s deacetylase-dependent suppression of *Scn4b* expression, and *Scn4b’s* selective expression in NAc D1- and D2-MSNs, and its reported localization in unmyelinated MSN projections to the ventral pallidum and substantia nigra (28) led us to explore SCN4B’s role in cued cocaine seeking. Hypothesized to function as an open channel blocker in neurons that express it (30,33), SCN4B is a single-pass transmembrane protein that covalently associates with pore-forming sodium channel alpha subunits. Upon membrane depolarization, the SCN4B intra-cellular domain is thought to enter the pore and block sodium ion flow, which prevents the channel from inactivating and, following membrane repolarization and the voltage-dependent removal of SCN4B from the channel pore, a resurgent sodium current flows and facilitates another AP. The SCN4B-regulated resurgent current facilitates high-frequency firing in cerebellar Purkinje cells and increases AP propagation fidelity in the unmyelinated projections of the striatum (28,30). However, *Scn4b* does not appear to be required for the expression of resurgent sodium current (31,56) and may enhance AP frequency and fidelity through a different mechanism. We showed here that HDAC5 reduces expression of *Scn4b* mRNA, and reducing *Scn4b* expression limits NAc MSN firing rate. Similar to effects of HDAC5–3SA, the reduction of NAc MSN firing rate by AAV2-shScn4b increases AP amplitude accommodation and decreases the time to depolarization block. Precisely how SCN4B regulates NAc MSN firing rate will be an important future question.

NAc HDAC5 limits cue-reinstated drug seeking through a key function during acquisition of drug self-administration (9). In this study, HDAC5-3SA in the NAc reduced *Scn4b* mRNA expression and MSN intrinsic excitability in drug-naïve animals, suggesting that HDAC5/SCN4B-dependent regulation of NAc MSN firing rate influences the synaptic plasticity that underlies the formation of drug-cue/context associations. While the relevant form of drug-induced plasticity is not yet clear, it is intriguing to note that SCN4B is required for spike timing–dependent long-term depression in NAc MSNs through the facilitation of backpropagating APs (29). SCN4B may support a NAc MSN AP frequency needed to strengthen excitatory inputs that mediate drug-cue, but not natural reward-cue, associations. Moreover, SCN4B is important for AP propagation fidelity in unmyelinated projections of the striatum to the substantia nigra pars reticulata (28). The ventral pallidum is a major target of NAc MSNs and is critical for cue-induced drug seeking (57), and SCN4B function may support NAc MSN→ventral pallidum synapse plasticity needed for cue-reinstated drug seeking.

## Conclusions

Our findings provide new insights into the molecular mechanism of HDAC5’s limiting of relapse-like behavior and, more broadly, provide an update regarding the role of intrinsic enzymatic activity in class IIa HDAC function. This study positions NAc MSN intrinsic excitability as a major factor underlying the establishment of lasting drug-cue associations and reveals the sodium channel modulator SCN4B to be a critical component supporting NAc MSN firing rate and relapse-like cocaine seeking. Targeting SCN4B and/or uncovering the mechanisms by which reduced MSN excitability limits drug-seeking behavior could lead to improved therapeutics to limit relapse vulnerability in patients with an SUD.

## Supplementary Material

1

2

3

Supplementary material cited in this article is available online at https://doi.org/10.1016/j.biopsych.2025.01.027.

## Figures and Tables

**Table:** Key resource table

Resource Type	Specific Reagent or Resource	Source or Reference	Identifiers	Additional Information
Add additional rows as needed for each resource type	Include species and sex when applicable.	Include name of manufacturer, company, repository, individual, or research lab. Include PMID or DOI for references; use “this paper” if new.	Include catalog numbers, stock numbers, database IDs or accession numbers, and/or RRIDs. RRIDs are highly encouraged; search for RRIDs at https://scicrunch.org/resources.	Include any additional information or notes if necessary.
Antibody	Alexa Fluor 488 AffiniPure Donkey anti-Chicken	Jackson ImmunoResearch Labs	Cat#: 703-545-155; RRID:AB_2340375	
Antibody	Alexa Fluor 594 AffiniPure Donkey anti-Mouse	Jackson ImmunoResearch Labs	Cat#: 715-585-150; RRID:AB_2340854	
Antibody	chicken anti-GFP	Aves Labs	Cat# GFP-1020; RRID:AB_10000240	
Antibody	Donkey anti-Mouse IgG, Alexafluor 488 conjugated	Thermo Fisher Scientific	Cat#: A-21202; RRID:AB_141607	
Antibody	IRDye 680RD goat anti-mouse	LI-COR Biosciences	Cat# 926-68070; RRID:AB_10956588	
Antibody	IRDye 680RD goat anti-rabbit	LI-COR Biosciences	Cat#: 926-68071; RRID:AB_10956166	
Antibody	IRDye 680RDdonkey anti-chicken	LI-COR Biosciences	Cat#: 926-68075; RRID:AB_10974977	
Antibody	IRDye 800CW donkey anti-chicken	LI-COR Biosciences	Cat# 926-32218; RRID:AB_1850023	
Antibody	IRDye 800CW goat anti-mouse	LI-COR Biosciences	Cat#: 926-32210; RRID:AB_621842	
Antibody	IRDye 800CW goat anti-rabbit	LI-COR Biosciences	Cat#: 926-32211; RRID:AB_621843	
Antibody	mouse anti-FLAG M2	Sigma-Aldrich	Cat# F1804; RRID:AB_262044	
Antibody	mouse anti-Scn4b N168/6	Antibodies Incorporated	Cat#: 75-198; RRID:AB_2301402	
Antibody	Rabbit anti-HA	Sigma-Aldrich	Cat# H6908; RRID:AB_260070	
Cell Line	HEK293T	ATCC	Cat# CRL-3216; RRID:CVCL_0063	
Cell Line	Rat Primary Striatal Neurons	This Paper	N/A	
Chemical Compound or Drug	Aquamount	Epredia	Cat# 14-390-5	
Chemical Compound or Drug	B27	Thermo Fisher Scientific	Cat# 17-504-044	
Chemical Compound or Drug	Brevital	Par Pharmaceutical	Cat# 42023010501	
Chemical Compound or Drug	Cefazolin	MWI Animal Health	Cat# MWI 008902	
Chemical Compound or Drug	Cocaine hydrochloride	RTI International	N/A	NIDA Drug Inventory Supply and Control System
Chemical Compound or Drug	cOmplete Mini EDTA-free protease inhibitor	Roche	Cat# 11836170001	
Chemical Compound or Drug	DMEM	Thermo Fisher Scientific	Cat# 11-960-044	
Chemical Compound or Drug	EZ View Anti-Flag M2 Affinity Gel	Millipore	Cat# F2426; RRID:AB_2616449	
Chemical Compound or Drug	EZ View Anti-HA M2 Affinity Gel	Millipore	Cat# E6779; RRID:AB_10109562	
Chemical Compound or Drug	FBS	Thermo Fisher Scientific	Cat# A3840001	
Chemical Compound or Drug	Heparin Lock Flush	Medline	Cat# 63323-545-05	
Chemical Compound or Drug	Hoescht 33342	Invitrogen	Cat# H3570	
Chemical Compound or Drug	iodoacetamide	Thermo Fisher Scientific	Cat# A39271	
Chemical Compound or Drug	Ketofen	MWI Animal Health	Cat# MWI 002800	
Chemical Compound or Drug	L-Glutamine	Sigma	Cat# G7513	
Chemical Compound or Drug	n-ethylmaleimide	Sigma	Cat# E3876	
Chemical Compound or Drug	Neurobasal	Thermo Fisher Scientific	Cat# 21-103-049	
Chemical Compound or Drug	Papain	Worthington	Cat# LS003126	
Chemical Compound or Drug	Pen-Strep	Millipore Sigma	Cat# P0781	
Chemical Compound or Drug	Porcine trypsin	Sigma	Cat# T4799	
Chemical Compound or Drug	Prolong Gold	Invitrogen	Cat# P36930	
Chemical Compound or Drug	Qiazol	Qiagen	Cat# 79306	
Chemical Compound or Drug	TCEP	Sigma	Cat# 646547	
Chemical Compound or Drug	TCS Lock	Access Technologies	Cat# NC0331668	
Commercial Assay Or Kit	HDAC5 Flurogenic Assay Kit	BPS BioScience	Cat# 50065	
Commercial Assay Or Kit	miRNeasy Mini	Qiagen	Cat# 217004	
Commercial Assay Or Kit	Superscript 3	Invitrogen	Cat# 18080044	
Deposited Data; Public Database	AlphaFold	PMID: 34265844; 34791371	RRID: SCR_023662	
Deposited Data; Public Database	HDAC4 Crystal Structure	PMID: 26819662	PDB ID: 5A2S	
Deposited Data; Public Database	HDAC8 Crystal Structure	PMID: 17721440	PDB ID: 2V5W	
Organism/Strain	Long-Evans Rats	Charles River	RRID: RGD_2308852	
Organism/Strain	Sprague-Dawley Rats	Charles River	RRID: RGD_734476	
Peptide, Recombinant Protein	PepM	Biosynth	N/A	[Cyc(3,5)]H2N-HQCMCGNTHVHPEHAGR^-OH
Peptide, Recombinant Protein	PepMox	Biosynth	N/A	[Cyc(3,5)]H2N-HQC(MetO)CGNTHVHPEHAGR^-OH
Recombinant DNA	AAV-CMV-3XFLAG-HDAC5-C696S	This Paper	N/A	
Recombinant DNA	AAV-CMV-3XFLAG-HDAC5-C698S	This Paper	N/A	
Recombinant DNA	AAV-CMV-3XFLAG-HDAC5-D870N	This Paper	N/A	
Recombinant DNA	AAV-CMV-FLAG-HDAC4	This Paper	N/A	
Recombinant DNA	AAV-CMV-HDAC3-HA	This Paper	N/A	
Recombinant DNA	AAV-CMV-MEF2C-HA	This Paper	N/A	
Recombinant DNA	MRE-mCherry	PMID: 27779093	N/A	
Recombinant DNA, Viral Strain	AAV-CMV-3XFLAG-HDAC5-3SA	Plasmid:This Paper, Virus: University of South Carolina Viral Vector Core	N/A	
Recombinant DNA, Viral Strain	AAV-CMV-3XFLAG-HDAC5-3SA-CSCS	Plasmid:This Paper, Virus: University of South Carolina Viral Vector Core	N/A	
Recombinant DNA, Viral Strain	AAV-CMV-3XFLAG-HDAC5-CSCS	Plasmid:This Paper, Virus: University of South Carolina Viral Vector Core	N/A	
Recombinant DNA, Viral Strain	AAV-CMV-3XFLAG-HDAC5-WT	Plasmid:This Paper, Virus: University of South Carolina Viral Vector Core	N/A	
Recombinant DNA, Viral Strain	AAV-GFP	Plasmid PMID: 28957664, Virus: University of South Carolina Viral Vector Core	N/A	
Recombinant DNA, Viral Strain	AAV-shLuciferase	This Paper	N/A	
Recombinant DNA, Viral Strain	AAV-shMEF2A	Plasmid PMID: 18760698, Virus: University of South Carolina Viral Vector Core	N/A	
Recombinant DNA, Viral Strain	AAV-shMEF2D	Plasmid PMID: 18760698, Virus: University of South Carolina Viral Vector Core	N/A	
Recombinant DNA, Viral Strain	AAV-shScn4b	Plasmid: This Paper, Virus: University of South Carolina Viral Vector Core	N/A	
Sequence-Based Reagent	RT-qPCR Primer: beta-tubulin FWD	This Paper	N/A	5’-CGACAATGAAGCCCTCTACGAC-3’
Sequence-Based Reagent	RT-qPCR Primer: beta-tubulin RVS	This Paper	N/A	5’-ATGGTGGCAGACACAAGGTGGTTG-3’
Sequence-Based Reagent	RT-qPCR Primer: *Mef2a* FWD	This Paper	N/A	5’-TCAAGCCACACAACCTCTTG-3’
Sequence-Based Reagent	RT-qPCR Primer: *Mef2a* RVS	This Paper	N/A	5’-CAGCATTCCAGGGGAAGTAA-3’
Sequence-Based Reagent	RT-qPCR Primer: *Mef2d* FWD	This Paper	N/A	5’-CAGCAGCCAGCACTACAGAG-3’
Sequence-Based Reagent	RT-qPCR Primer: *Mef2d RVS*	This Paper	N/A	5’-GGCAGGGATGACCTTGTTTA-3’
Sequence-Based Reagent	RT-qPCR Primer: *Scn4b* FWD	This Paper	N/A	5’-GGGCTTTTGGGTCTCTTC-3’
Sequence-Based Reagent	RT-qPCR Primer: *Scn4b* RVS	This Paper	N/A	5’-GAGGTTCTCAAAGCCATAACA-3’
Software; Algorithm	AMBER20	PMID: 37805934	N/A	
Software; Algorithm	AxoGraphX v.1.8.0	AxoGraph	RRID:SCR_014284	
Software; Algorithm	Clampfit v.8.0	Molecular Devices	RRID:SCR_011323	
Software; Algorithm	MaxQuant v. 2.0.1.0	Max Planck Institute	N/A	
Software; Algorithm	MiniAnalysis Program v.6.0.9	Synaptosoft	RRID:SCR_002184	
Software; Algorithm	OpenMM	PMID: 38154096	N/A	
Software; Algorithm	Prism	GraphPad	RRID:SCR_002798	
