# Supplementary material for: Epigenetic Control of an Auxiliary Subunit of Voltage-Gated Sodium Channels Regulates the Strength of Drug-Cue Associations and Relapse-Like Cocaine Seeking
Source: Biol Psychiatry. Author manuscript; Available in PMC 2026 Apr 15. (PMC13078857; doi:10.1016/j.biopsych.2025.01.027)
Supplement: Supplementary file 1 [file NIHMS2158977-supplement-1.pdf]

SUPPLEMENTARY INFORMATION

Epigenetic Control of an Auxiliary Subunit of Voltage-Gated Sodium Channels Regulates the Strength of Drug–Cue Associations and Relapse-Like Cocaine Seeking

Wood *et al.*

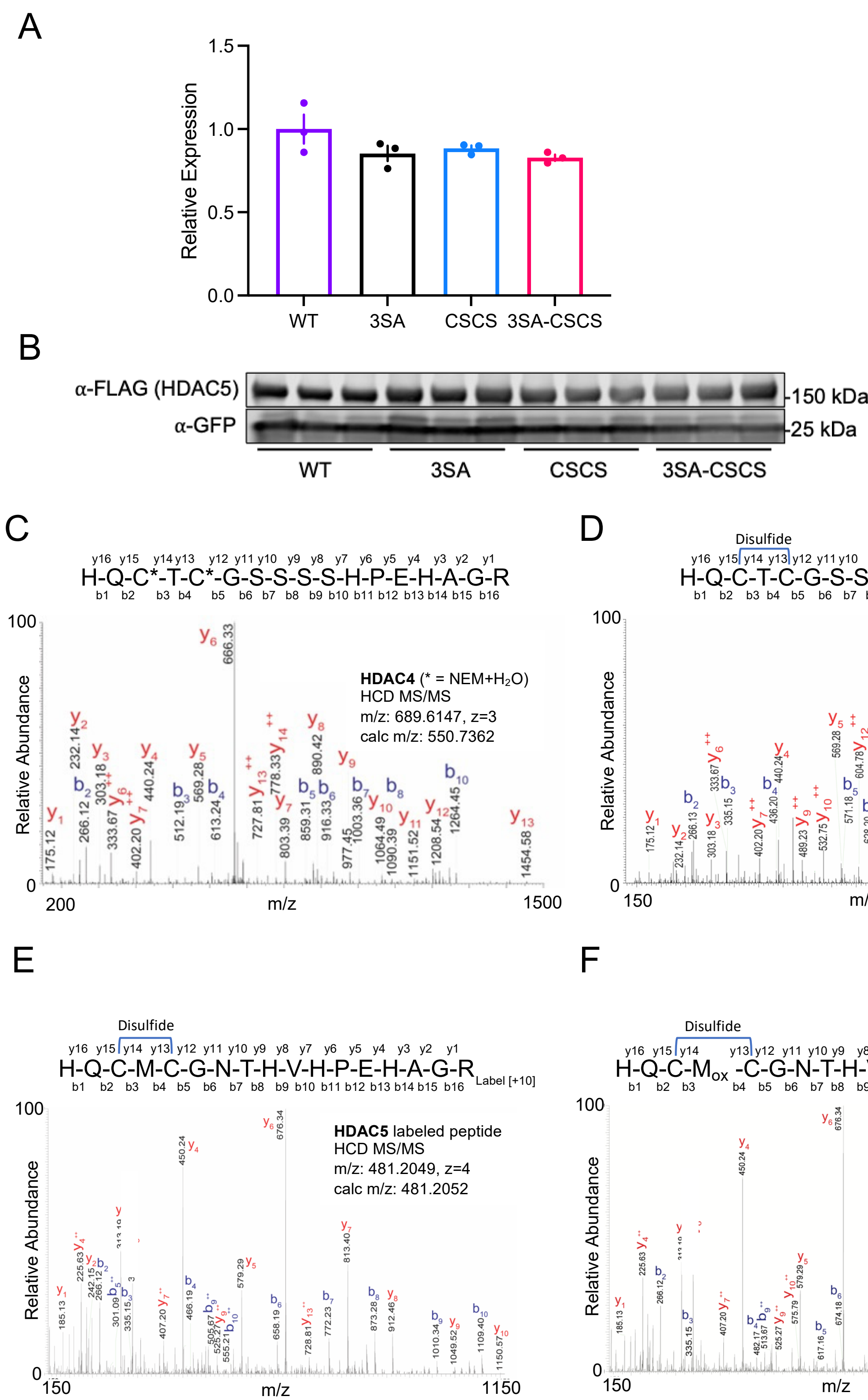

Figure S1.
(**A-B**) AAV-HDAC5 constructs were transfected in equal concentrations into HEK-293T cells and protein immunoblot was used to confirm equivalent expression of all constructs. (**A**) Densitometric quantification of HDAC5 mutant immunoblot (n = 3 all groups; One-way ANOVA; No difference between groups observed). (**B**) Representative immunoblot of α-FLAG (HDAC5) and α-GFP. (**C**) HEK-293T cell-derived HDAC4 MS2 spectrum consistent with NEM-alkylated (reduced) CXC cysteines. (**D**) HEK-293T cell-derived HDAC4 MS2 spectrum consistent with an intramolecular disulfide bond between the CXC cysteines. (**E-F**) MS2 spectra of arginine heavy-isotope labeled standards for SureQuant targeted analysis of NAc -derived HDAC5. Heavy-labeled HDAC5 CXC tryptic fragments were synthesized with unoxidized methionine 697 (**E**) and oxidized M697 (**F**). Data shown are mean ± S.E.M.; Each point represents one cell-culture well (**A**).

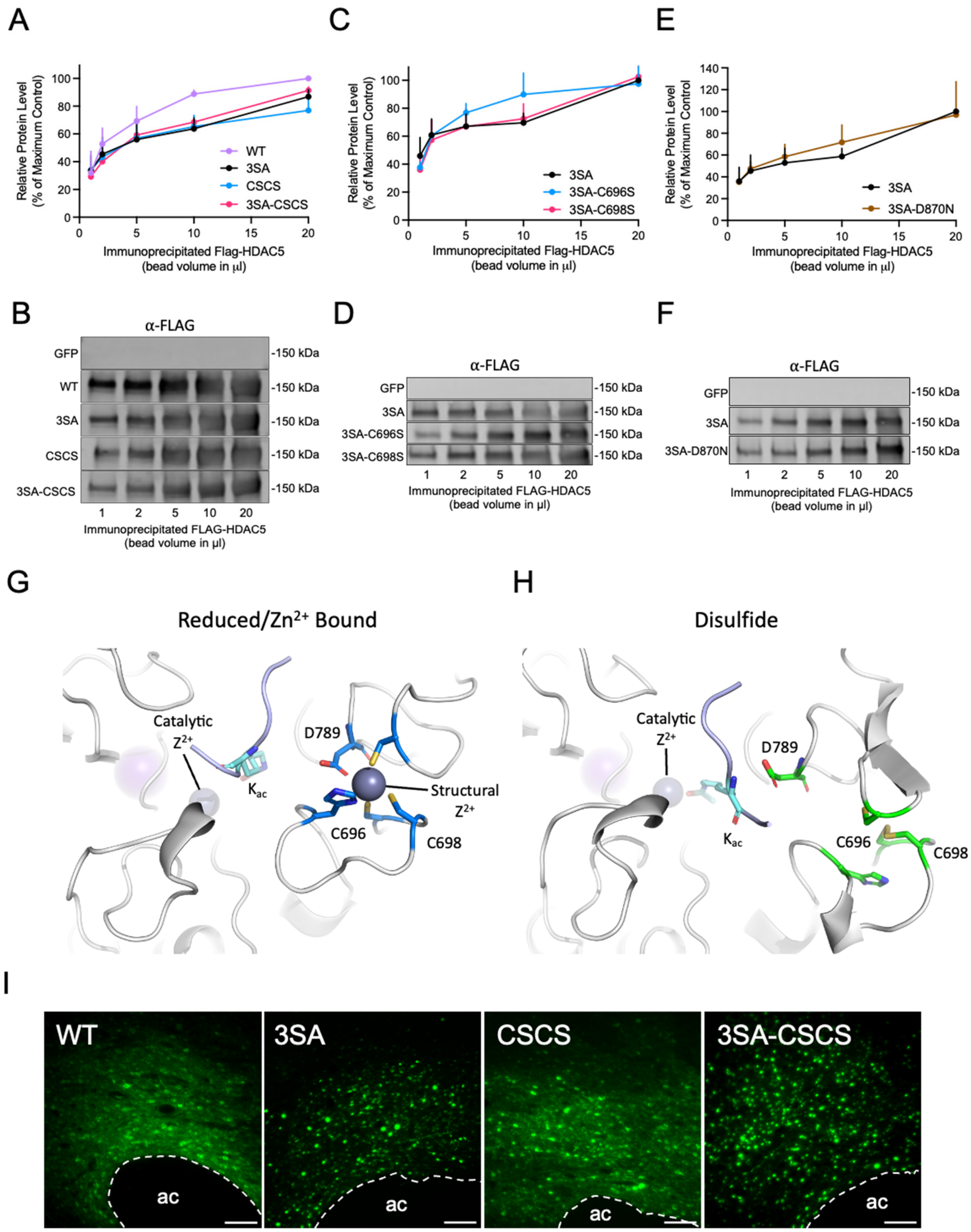

Figure S2.

(**A**) Densitometric quantification of HDAC5-CSCS mutant immunoblot of samples used in enzymatic activity assay (Two-Way RM ANOVA; No differences between groups observed). (**B**) Representative images of HDAC5-CSCS mutant immunoblot of samples used in enzymatic activity assay. (**C**) Densitometric quantification of HDAC5 single-cysteine mutant immunoblot of bead samples used in enzymatic activity assay (Two-Way RM ANOVA; No differences between groups observed). (**D**) Representative images of HDAC5 single-cysteine mutant immunoblot at bead volumes used in enzymatic activity assay. (**E**) Densitometric quantification of HDAC5 catalytic site mutant immunoblot of bead samples used in enzymatic activity assay (Two-Way RM ANOVA; No differences between groups observed). (**F**) Representative images of HDAC5 catalytic site mutant immunoblot at bead volumes used in enzymatic activity assay. (**G-H**) Structural comparison of modeled structural zinc-binding domain of HDAC5 without (**G**) and with (**H**) a disulfide bond between C696 and C698 (K_{ac}: H3K9ac peptide; Grey sphere: Zn^{2+}; Purple sphere: K^{+}). (**I**) Low magnification images of AAV2-HDAC5 expression in rat NAc following α-FLAG immunostaining (AC: anterior commissure; Scale bars: 100 µm).

Data shown are mean ± S.E.M. (**A**, **C**, **E**).

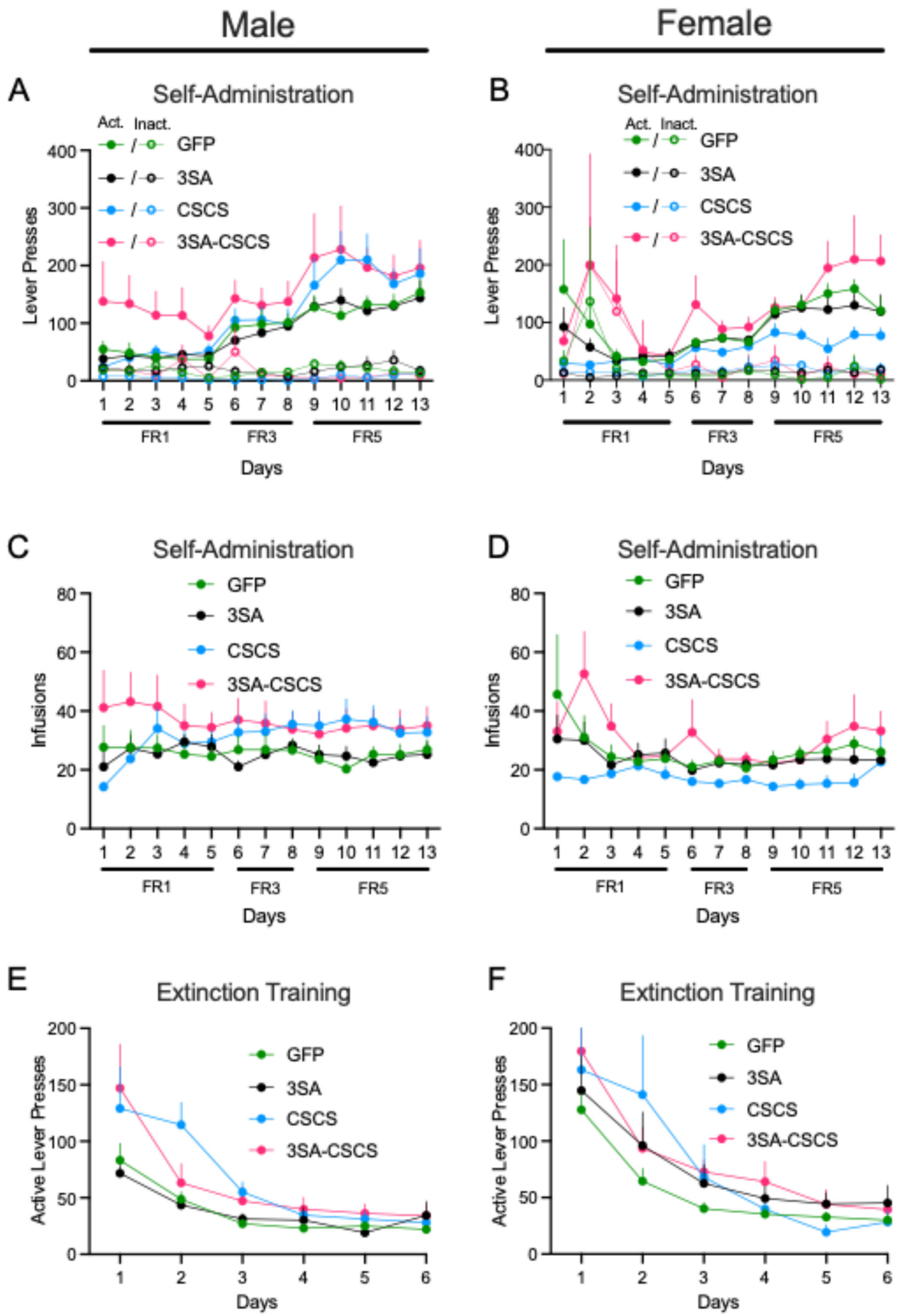

Figure S3.
(**A-F**) Cocaine intravenous self-administration data presented in Figure 3 disaggregated by sex. Male data on left showing lever presses (**A**) & cocaine infusions (**C**) during acquisition and active lever presses (**E**) during extinction. Female data on right showing lever presses (**B**) & cocaine infusions (**D**) during acquisition and active lever presses (**F**) during extinction. Data shown are mean ± S.E.M.

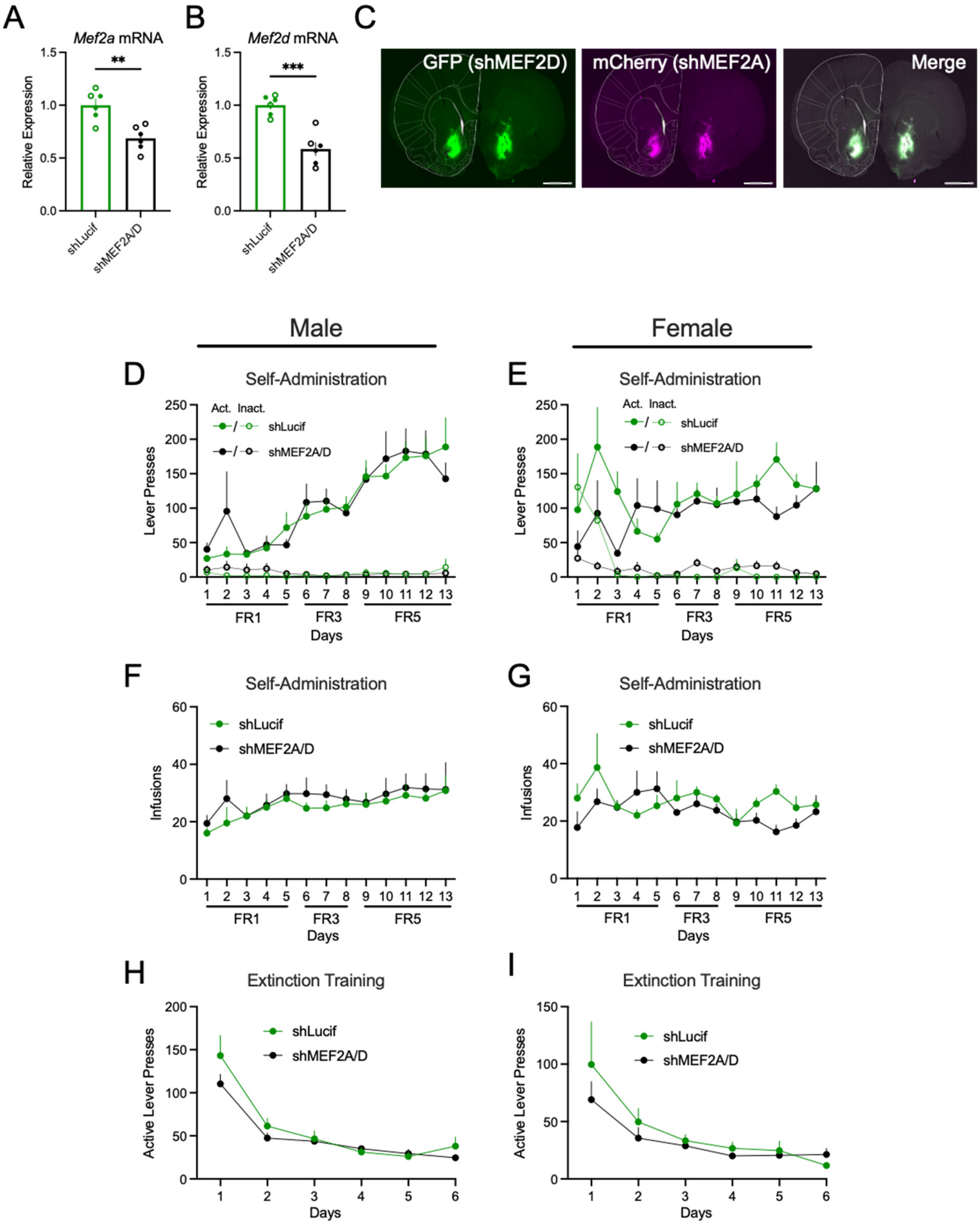

Figure S4.

(**A**) *Mef2a* mRNA expression levels as measured by qPCR. shMEF2A/D reduces *Mef2a* mRNA levels compared to shLuciferase controls (shLucif: n = 6 (3 male, 3 female), shMEF2A/D: n = 6 (3 male, 3 female); Unpaired two-tail t-test; **$p < 0.01$). (**B**) *Mef2d* mRNA expression levels as measured by qPCR. shMEF2A/D reduces *Mef2d* mRNA levels compared to shLuciferase controls (shLucif: n = 6 (3 male, 3 female), shMef2a/d: n = 6 (3 male, 3 female), Unpaired two-tail t-test; ***$p < 0.001$). (**C**) Representative images of AAV2-shMEF2A and AAV2-shMEF2D viral co-expression (Scale Bar: 2000 um). (**D-I**) Cocaine intravenous self-administration data presented in Figure 4 disaggregated by sex. Male data on left showing lever presses (**D**) & cocaine infusions (**F**) during acquisition and active lever presses (**H**) during extinction. Female data on right showing lever presses (**E**) & cocaine infusions (**G**) during acquisition and active lever presses (**I**) during extinction. Data shown are mean ± S.E.M. (**A-B**, **D-I**); Each point represents one rat (**A-B**); Closed circles: male, open circles: female (**A-B**).

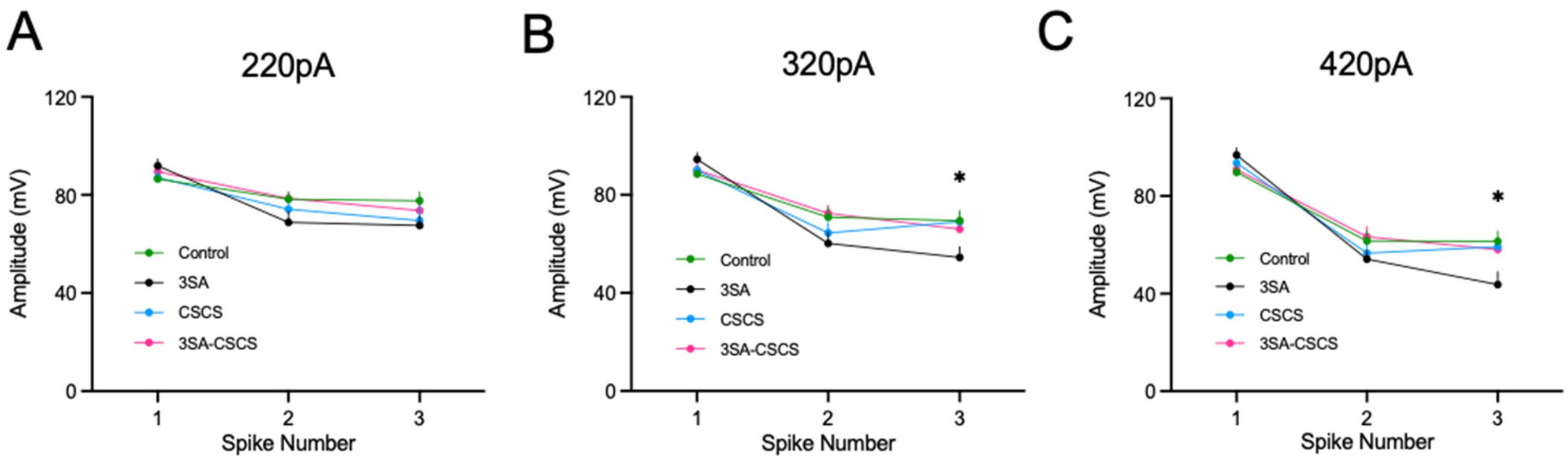

Figure S5.
(**A-C**) Amplitudes of first three spikes at 220 (**A**), 320 (**B**) and 420 pA (**C**) current injections. Control: *n* = 22 cells/4 rats, 3SA: *n* = 19 cells/4 rats, CSCS: *n* = 17 cells/4 rats, 3SA-CSCS: *n* = 22 cells/4 rats. Data analyzed by Two-Way RM ANOVA w/ Fishers LSD Post-Hoc. *$p < 0.05$ 3SA vs. Control pairwise comparison. Data shown are mean ± S.E.M.

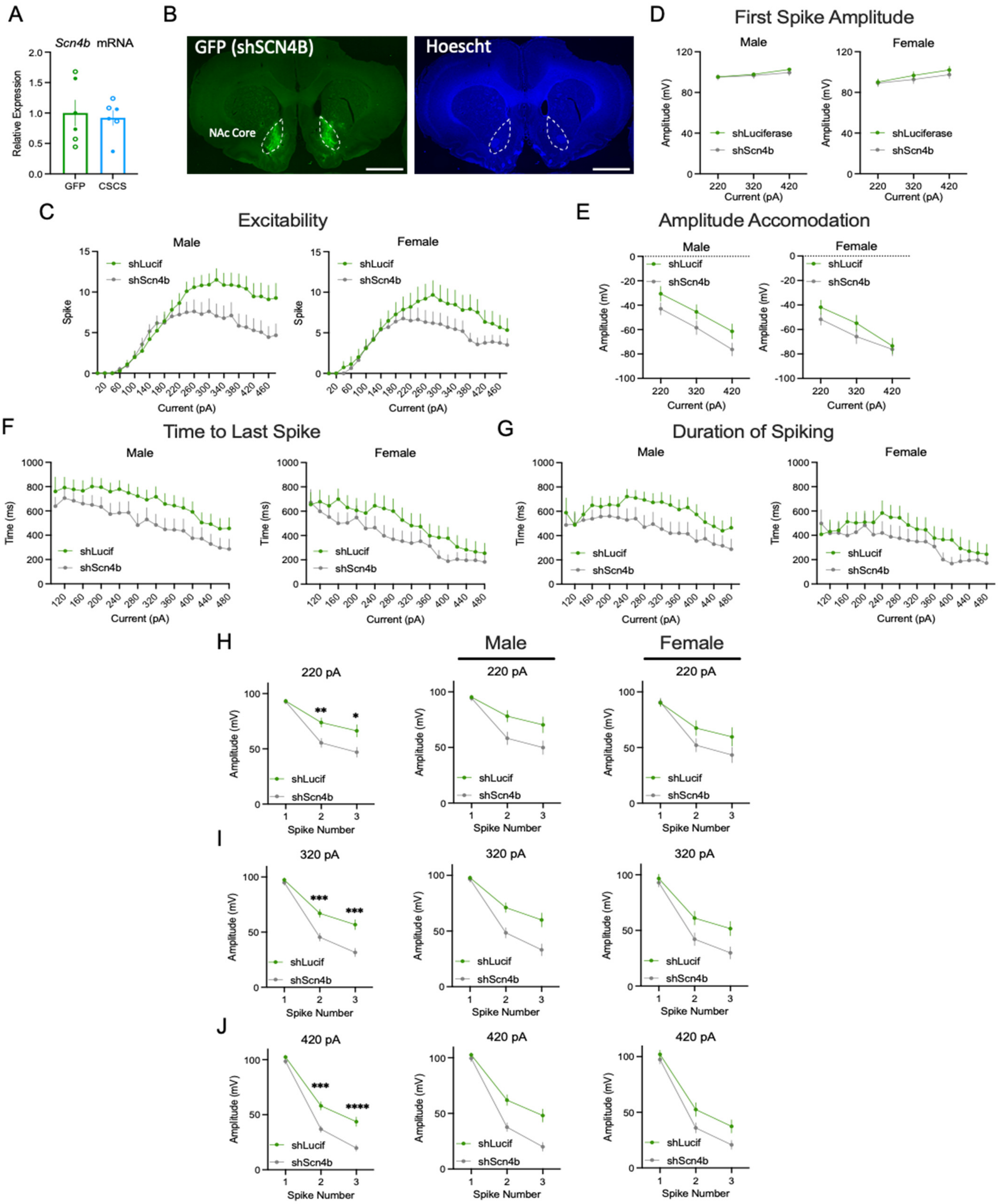

Figure S6.

(**A**) *Scn4b* mRNA levels in rat NAc following HDAC5-CSCS expression. (Unpaired two-tailed t-test; No differences between groups observed) (**B**) Representative images of AAV2-shScn4b virus expression in NAc (scale bar: 2000 um). (**C-G**) Electrophysiological data presented in Figure 6 disaggregated by sex (not statistically analyzed). (**H-J Left**) Amplitudes of first three spikes at 220 (**G**), 320 (**H**), and 420 pA (**I**) (Mixed-Effect Model (REML) w/ Šídák's post-hoc of aggregated data; $*p < 0.05$, $**p < 0.01$, $***p < 0.001$, $****\ p < 0.0001$ shLucif vs. shScn4b pairwise comparisons). (**H-J Middle, Right**) Data disaggregated by sex (*middle*: males; *right*: females; not statistically analyzed). Data shown are mean ± S.E.M (**A**, **C-J**).

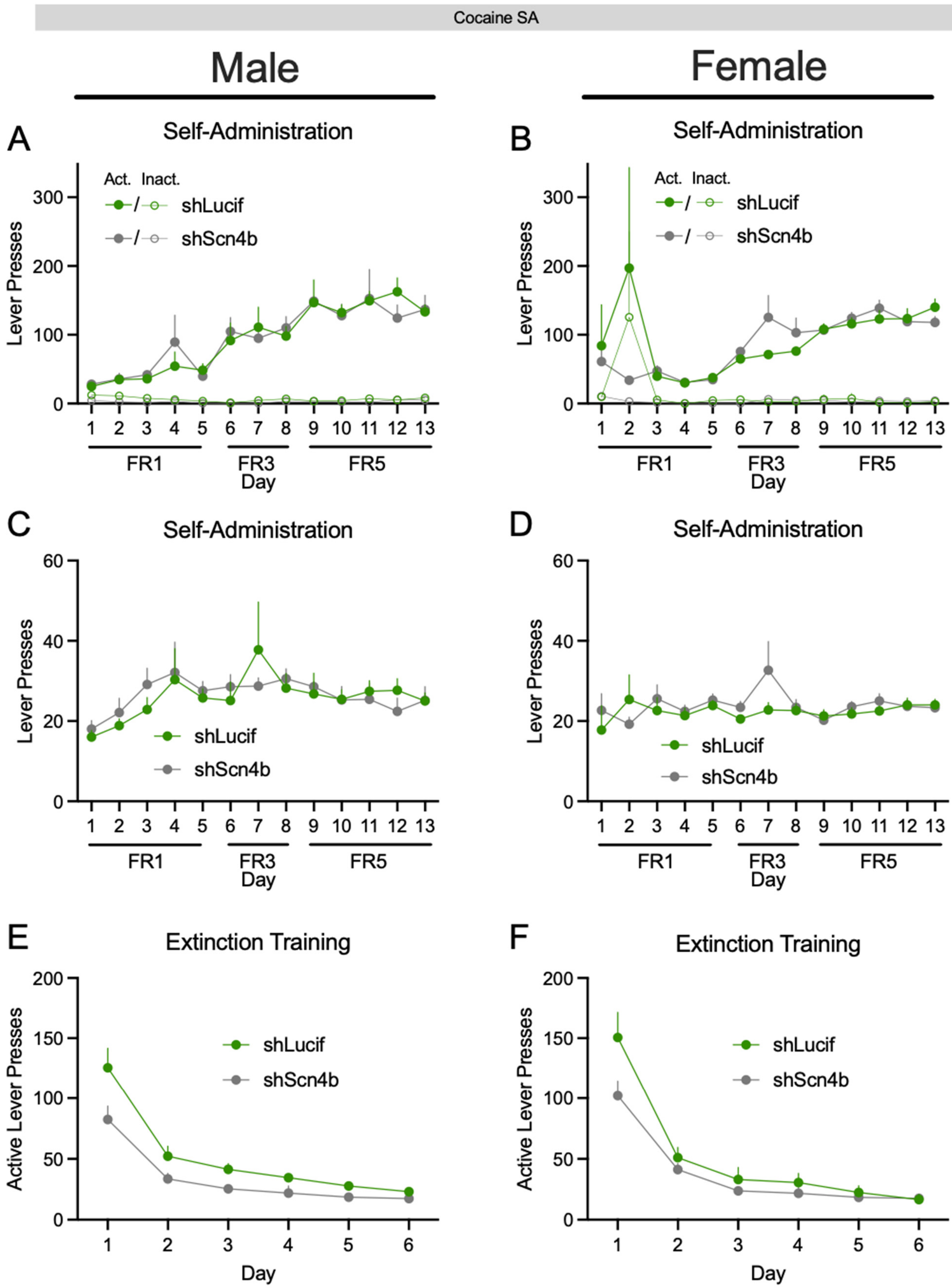

Figure S7.
(**A-F**) Cocaine intravenous self-administration data presented in Figure 7 disaggregated by sex. Male data on left showing lever presses (**A**) & cocaine infusions (**C**) during acquisition and active lever presses (**E**) during extinction. Female data on right showing lever presses (**B**) & cocaine infusions (**D**) during acquisition and active lever presses (**F**) during extinction. Data shown are mean ± S.E.M. Not statistically analyzed.

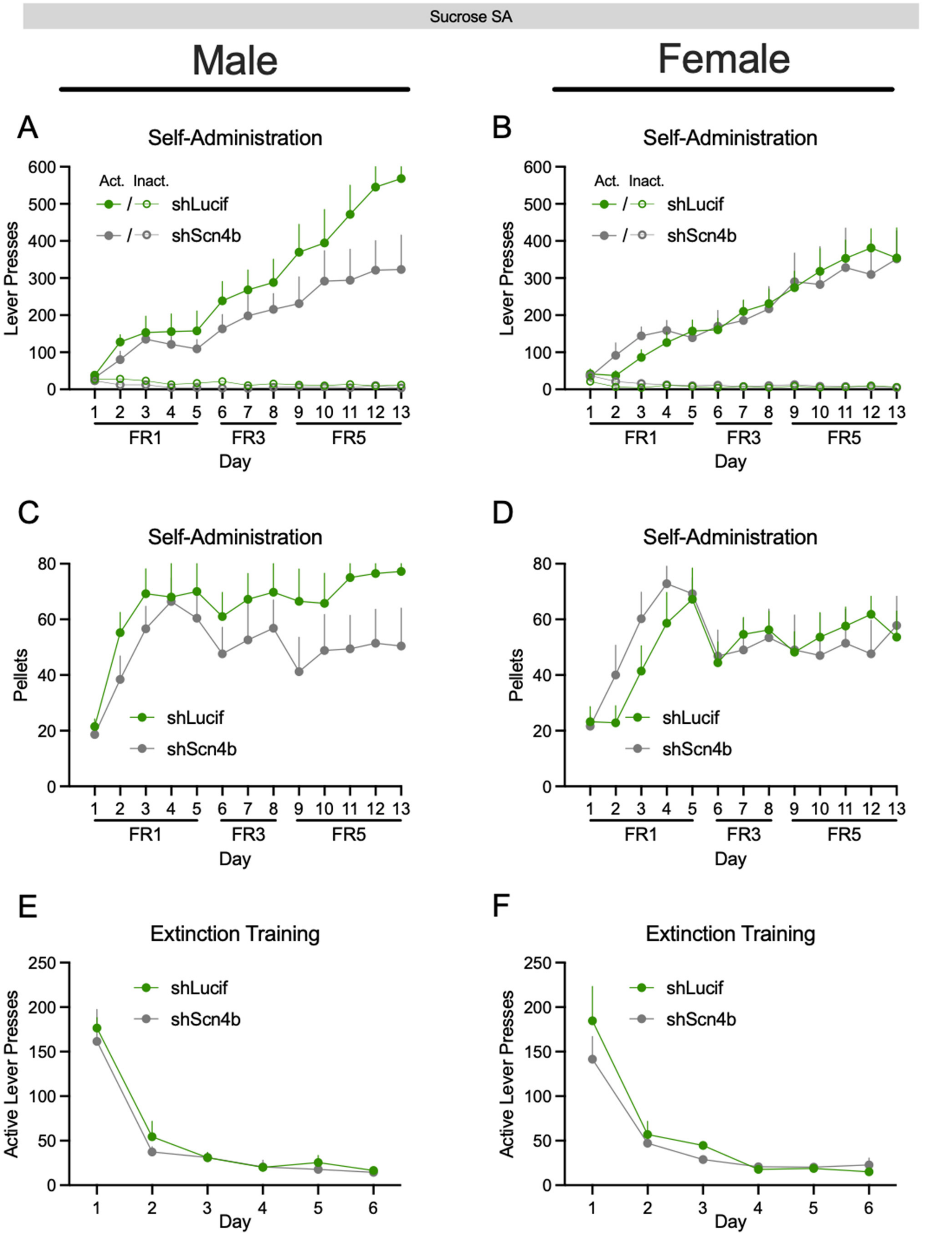

Figure S8.

(**A-F**) Sucrose self-administration data presented in Figure 7 disaggregated by sex. Male data on left showing lever presses (**A**) & sucrose pellets (**C**) during acquisition and active lever presses (**E**) during extinction. Female data on right showing lever presses (**B**) & sucrose pellets (**D**) during acquisition and active lever presses (**F**) during extinction. Data shown are mean ± S.E.M. Not statistically analyzed.

Table S1.
HDAC4 Experiment 1.

Sequence	Modifications	MQ Intensity	Score	MS/MS Count	Mass		RT (min)	% of total	MS/MS scan number
HQCTCGSSSSHPEHAGR	2 NEM+water	30,937,000,000	193	49	2065.83	Free thiol	13.67	58.2	9614
HQCTCGSSSSHPEHAGR	NEM; NEM+water	14,387,000,000	265	48	2047.82	Free thiol	17.20	27.0	11978
HQCTCGSSSSHPEHAGR	Sulfide(C); Cys->Dha(C)	4,174,600,000	233	6	1777.70	Disulfide	6.99	7.8	4834
HQCTCGSSSSHPEHAGR	Cys->Dha(C); NEM+water	1,905,200,000	186	9	1888.79		8.53	3.6	6065
HQCTCGSSSSHPEHAGR	2 NEM	882,360,000	308	5	2029.81	Free thiol	20.84	1.7	15590
HQCTCGSSSSHPEHAGR	NEM+water	302,600,000	115	11	1922.77		11.37	0.6	9253
HQCTCGSSSSHPEHAGR	Mono oxidation (sulfenic)(C); NEM	216,970,000	126	21	1920.76		10.01	0.4	5665
HQCTCGSSSSHPEHAGR	Tri oxidation (sulfonic)(C); NEM+water	187,690,000	91	5	1970.76		9.05	0.4	6809
HQCTCGSSSSHPEHAGR	Tri oxidation (sulfonic)(C); NEM	107,420,000	116	3	1952.75		14.51	0.2	9990
HQCTCGSSSSHPEHAGR	Di oxidation (sulfinic)(C); NEM	99,856,000	106	5	1936.75		14.24	0.2	10058

NEM = N-ethylmaleimide
Cys->Dha = Loss of SH on cysteine (C) with Sulfide (C) implies an intrapeptide disulfide bond is present

Table S2.
HDAC5 Experiment 1

Sequence	Modifications	MQ Intensity	Score	MS/MS Count	Mass		RT (min)	% of total	MS/MS scan number
HQCMCGNTHVHPEHAGR	Sulfide(C); Cys->Dha(C)	21,533,000,000	212.7	20	1910.78	Disulfide	15.51	43.4	11747
HQCMCGNTHVHPEHAGR	2 NEM+water	13,031,000,000	202.3	78	2198.92	Free thiol	25.95	26.3	18450
HQCMCGNTHVHPEHAGR	NEM; NEM+water	7,593,600,000	199.3	60	2180.91	Free thiol	28.68	15.3	23188
HQCMCGNTHVHPEHAGR	Oxidation (M); Sulfide(C); Cys->Dha(C)	1,954,200,000	157.8	28	1926.78	Disulfide	14.13	3.9	10827
HQCMCGNTHVHPEHAGR	Oxidation (M); 2 NEM+water	1,461,300,000	163.4	41	2214.91	Free thiol	21.73	2.9	16070
HQCMCGNTHVHPEHAGR	Oxidation (M); NEM	1,318,100,000	128.8	28	2053.84		21.31	2.7	19200
HQCMCGNTHVHPEHAGR	2 NEM	1,022,800,000	220.0	19	2162.89	Free thiol	30.99	2.1	25606
HQCMCGNTHVHPEHAGR	Cys->Dha(C); NEM+water	929,830,000	164.4	29	2021.87		19.58	1.9	16980
HQCMCGNTHVHPEHAGR	NEM+water	400,820,000	165.6	10	2055.86		25.72	0.8	20441
HQCMCGNTHVHPEHAGR	Oxidation (M); NEM; NEM+water	386,230,000	109.4	15	2196.90	Free thiol	23.64	0.8	17305

NEM = N-ethylmaleimide
Cys->Dha = Loss of SH on cysteine (C) with Sulfide (C) implies an intrapeptide disulfide bond is present

Table S3.
HDAC4 Experiment 2

Sequence	Modifications	MQ Intensity	Score	MS/MS Count	Mass		RT (min)	% of Total	MS/MS scan number
HQCTCGSSSSHPEHAGR	NEM; NEM+Water	21,056,000,000	204	125	2047.82	Free thiol	12.55	48.31	9492
HQCTCGSSSSHPEHAGR	2 NEM	7,566,900,000	249	62	2029.81	Free thiol	15.07	17.36	19889
HQCTCGSSSSHPEHAGR	Sulfide(C);Cys->Dha(C)	6,095,000,000	137	22	1777.70	Disulfide	4.07	13.98	4203
HQCTCGSSSSHPEHAGR	2 NEM+Water	4,150,700,000	166	59	2065.83	Free thiol	7.97	9.52	6236
HQCTCGSSSSHPEHAGR	Cys->Dha(C); NEM	2,277,500,000	142	9	1870.78		5.95	5.23	5742
HQCTCGSSSSHPEHAGR	MonoOx(C) Sulfenic; NEM	1,063,300,000	112	11	1920.76		5.30	2.44	5316
HQCTCGSSSSHPEHAGR	Cys->Dha(C); NEM+Water	839,540,000	162	4	1888.79		5.80	1.93	5371
HQCTCGSSSSHPEHAGR	DiOx(C) Sulfinic; NEM	206,860,000	130	5	1936.75		6.26	0.47	6525
HQCTCGSSSSHPEHAGR	DiOx(C)Sulfinic; NEM+Water	206,010,000	121	4	1954.76		5.80	0.47	5568
HQCTCGSSSSHPEHAGR	TriOx(C) Sulfonic;NEM	126,310,000	125	10	1952.75		6.73	0.29	6481

NEM = N-ethylmaleimide
Cys->Dha = Loss of SH on cysteine (C) with Sulfide (C) implies an intrapeptide disulfide bond is present

Table S4.
HDAC5 Experiment 2

Sequence	Modifications	MQ Intensity	Score	MS/MS Count	Mass		RT (min)	% of Total	MS/MS scan number
HQCMCGNTHVHPEHAGR	Sulfide(C); Cys->Dha(C)	34,707,000,000	156	13	1910.78	Disulfide	6.11	31.81	6149
HQCMCGNTHVHPEHAGR	Oxidation (M); Sulfide(C); Cys->Dha(C)	21,977,000,000	121	12	1926.78	Disulfide	7.61	20.14	5665
HQCMCGNTHVHPEHAGR	NEM; NEM+Water	21,137,000,000	169	115	2180.91	Free thiol	24.78	19.37	28366
HQCMCGNTHVHPEHAGR	2 NEM+Water	16,470,000,000	115	91	2198.92	Free thiol	21.31	15.10	22998
HQCMCGNTHVHPEHAGR	2 NEM	4,872,400,000	162	23	2162.89	Free thiol	27.36	4.47	32890
HQCMCGNTHVHPEHAGR	Oxidation (M); 2 NEM+Water	3,336,200,000	114	76	2214.91	Free thiol	12.81	3.06	14256
HQCMCGNTHVHPEHAGR	Oxidation (M); NEM	2,451,500,000	99	41	2053.84		15.23	2.25	16799
HQCMCGNTHVHPEHAGR	Oxidation (M); NEM; NEM+Water	1,495,200,000	119	48	2196.90	Free thiol	18.39	1.37	16798
HQCMCGNTHVHPEHAGR	NEM+Water	1,327,600,000	59	4	2055.86		20.44	1.22	23607
HQCMCGNTHVHPEHAGR	Cys->Dha(C); NEM+Water	1,327,100,000	111	38	2021.87		14.47	1.22	16013

NEM = N-ethylmaleimide
Cys->Dha = Loss of SH on cysteine (C) with Sulfide (C) implies an intrapeptide disulfide bond is present

Table S5.
Protein Groups Data

To determine if HDAC4 and HDAC5 were loaded at similar amounts for LC-MS/MS: Protein Groups Data from files:	Protein IDs	Protein names	Gene names	Unique peptides	Sequence coverage [%]	Mol. weight [kDa]	Sequence length	Protein Intensity
H5 02032023	Q9UQL6	Histone deacetylase 5	HDAC5	126	80.9	121.98	1122	2.1365E+12
5WT (second) 07252023	Q9UQL6	Histone deacetylase 5	HDAC5	117	80.8	121.98	1122	3.173E+12
H4 02032023	P56524	Histone deacetylase 4	HDAC4	118	81.6	119.04	1084	2.353E+12
H4 second 07262023	P56524	Histone deacetylase 4	HDAC4	121	84.2	119.04	1084	3.0864E+12

Supplemental data for identification of HDAC5 peptide using stable isotope standard triggered parallel reaction monitoring (IS-PRM) using SureQuant acquisition mode (Thermo Scientific).

Stable Isotope Labeled Peptides of HDAC5	Spiked (fmol)	Retention Time (min)	Precursor Ion (m/z), z=4	Trigger Threshold	Product ion	Product Ions (m/z)
[Cyc(3,5)]HQCMCGNTHVHPEHAGR*	250	12.4	481.2052	100,000		
					y^4	450.2447
					y^5	579.2870
					y^6	676.3401
					y^7	813.3990
					b^8	873.2814
					y^8	912.4674
[Cyc(3,5)]HQCM(ox)CGNTHVHPEHAGR*	125	10.0	485.2040	103,000		
					y^4	450.2447
					y^5	579.2870
					y^6	676.3401
					y^7	813.3990
					b^8	889.2750
					y^8	912.4674

(Cyc(3,5) indicates disulfide linkage between positions 3 and 5. *R is heavy, stable isotope encoded arginine ($^{13}C_6$, $^{15}N_4$). M(ox) indicates oxidized methionine. Spontaneous methionine oxidation of the unoxidized, synthetic peptide was observed. SureQuant acquisition mode was used to acquire data on the immunoprecipitated peptides with calculated monoisotopic m/z 478.7032 and 482.7019.

Rat NAc-Immunoprecipitated HDAC5 peptide: HQC(Cys->Dha)M(ox)C(Sulfide)GNTHVHPEHAGR

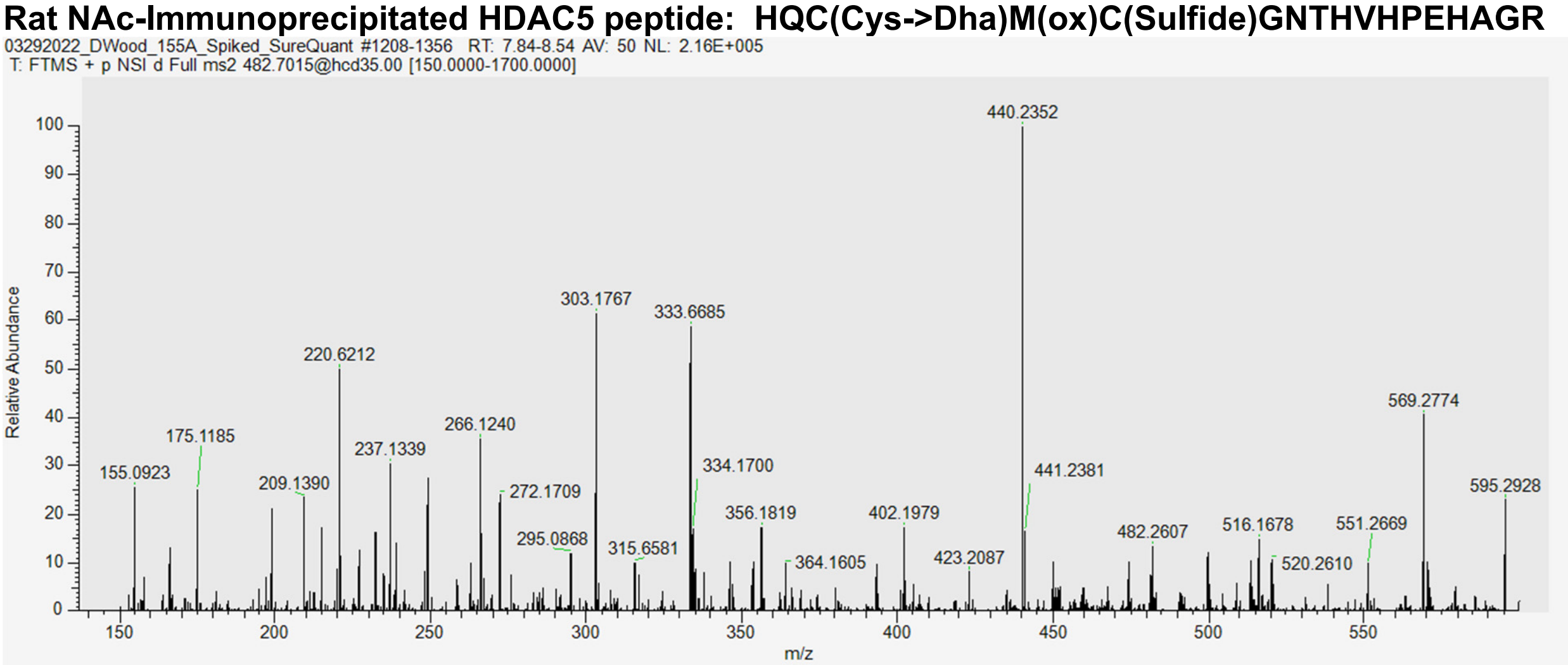

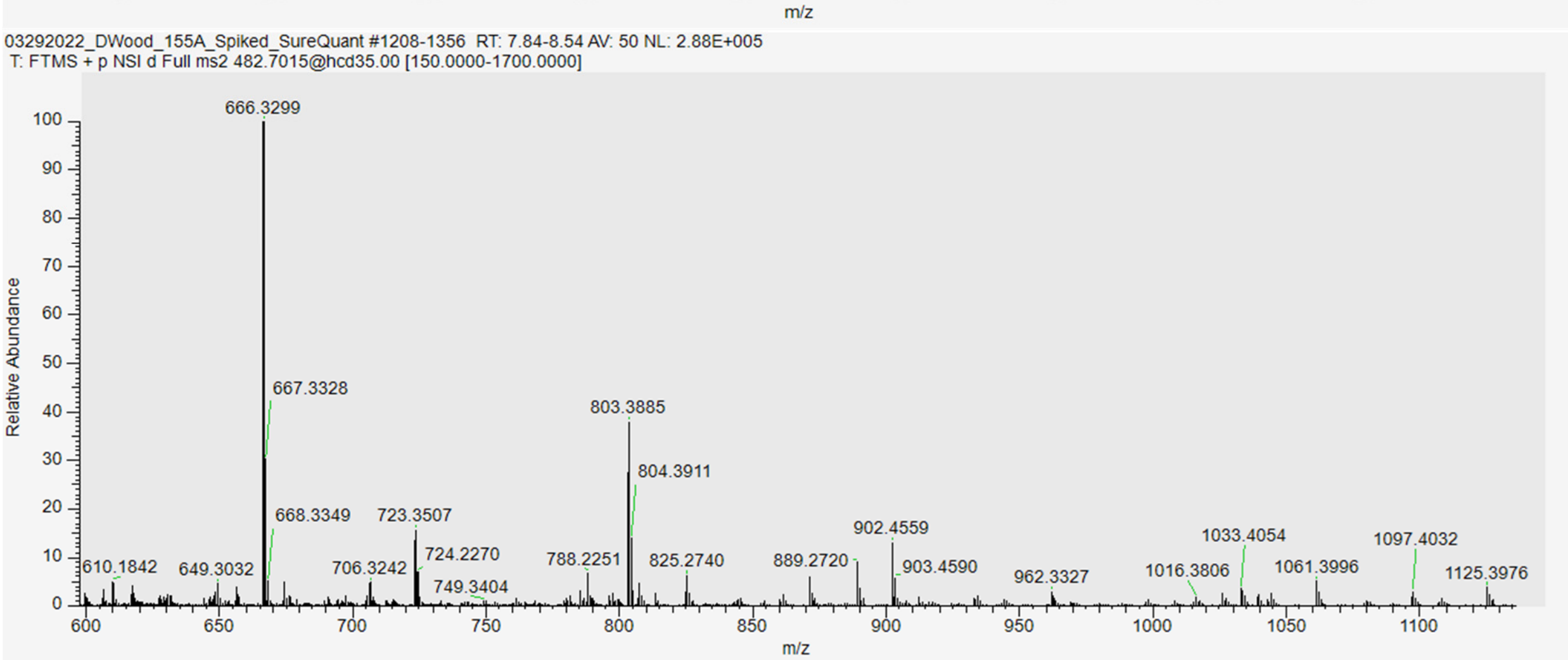

HQC(cys->dha)M(ox)C(sulfide)GNTHVHPEHAGR						
**MH^{+1}(av)**	**MH^{+1}(mono)**	**MH^{+2}(av)**	**MH^{+2}(mono)**	**MH^{+3}(av)**	**MH^{+3}(mono)**	**MH^{+4}(mono)**
1929.141	1927.8	965.07	964.3965	643.72	643.27	482.7019
**b**	**b^{+2}**				**y**	**y^{+2}**
---	---	1	**H**	17	---	---
266.1248	133.566	2	**Q**	16	1790.73	895.867
335.1462	168.077	3	**C(cys->dha)**	15	1662.67	831.838
482.1816	241.595	4	**M(ox)**	14	1593.65	797.327
617.1629	309.085	5	**C(sulfide)**	13	1446.61	723.809
674.1843	337.596	6	**G**	12	1311.63	656.319
788.2273	394.617	7	**N**	11	1254.61	627.808
889.275	445.141	8	**T**	10	1140.57	570.787
1026.3339	513.671	9	**H**	9	1039.52	520.263
1125.4023	563.205	10	**V**	8	902.459	451.733
1262.4612	631.734	11	**H**	7	803.391	402.199
1359.514	680.261	12	**P**	6	666.332	333.67
1488.5565	744.782	13	**E**	5	569.279	285.143
1625.6155	813.311	14	**H**	4	440.236	220.622
1696.6526	848.83	15	**A**	3	303.178	152.092
1753.674	877.341	16	**G**	2	232.14	116.574
---	---	17	**R**	1	175.119	88.0631

Loss from M(ox)				b4	b5	b6	b7	b8	b9	b10	b11
b-$SOCH_4$	---	---	---	418.183	553.165	610.186	724.229	825.277	962.336	1061.4	1198.46

Heavy Labeled Synthetic peptide:
HQC(cys->dha)M(ox)C(sulfide)GNTHVHPEHAGR(Label:13C(6)15N(4))

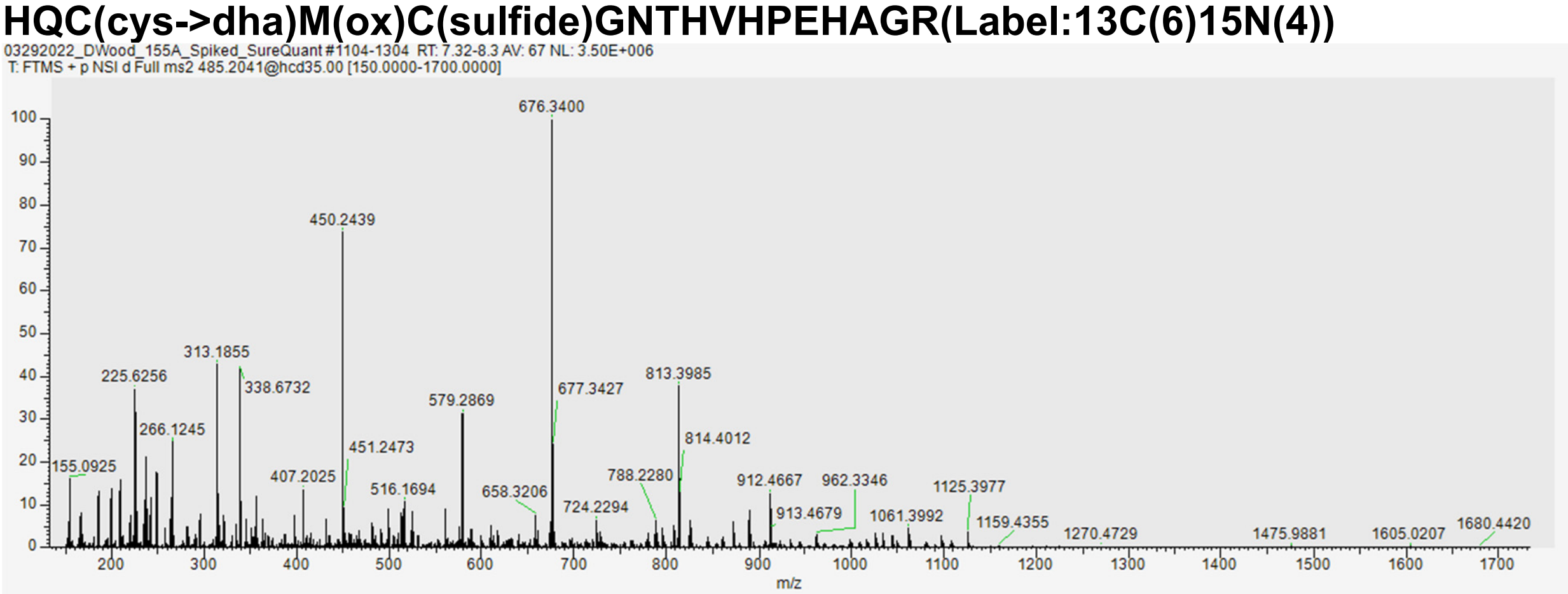

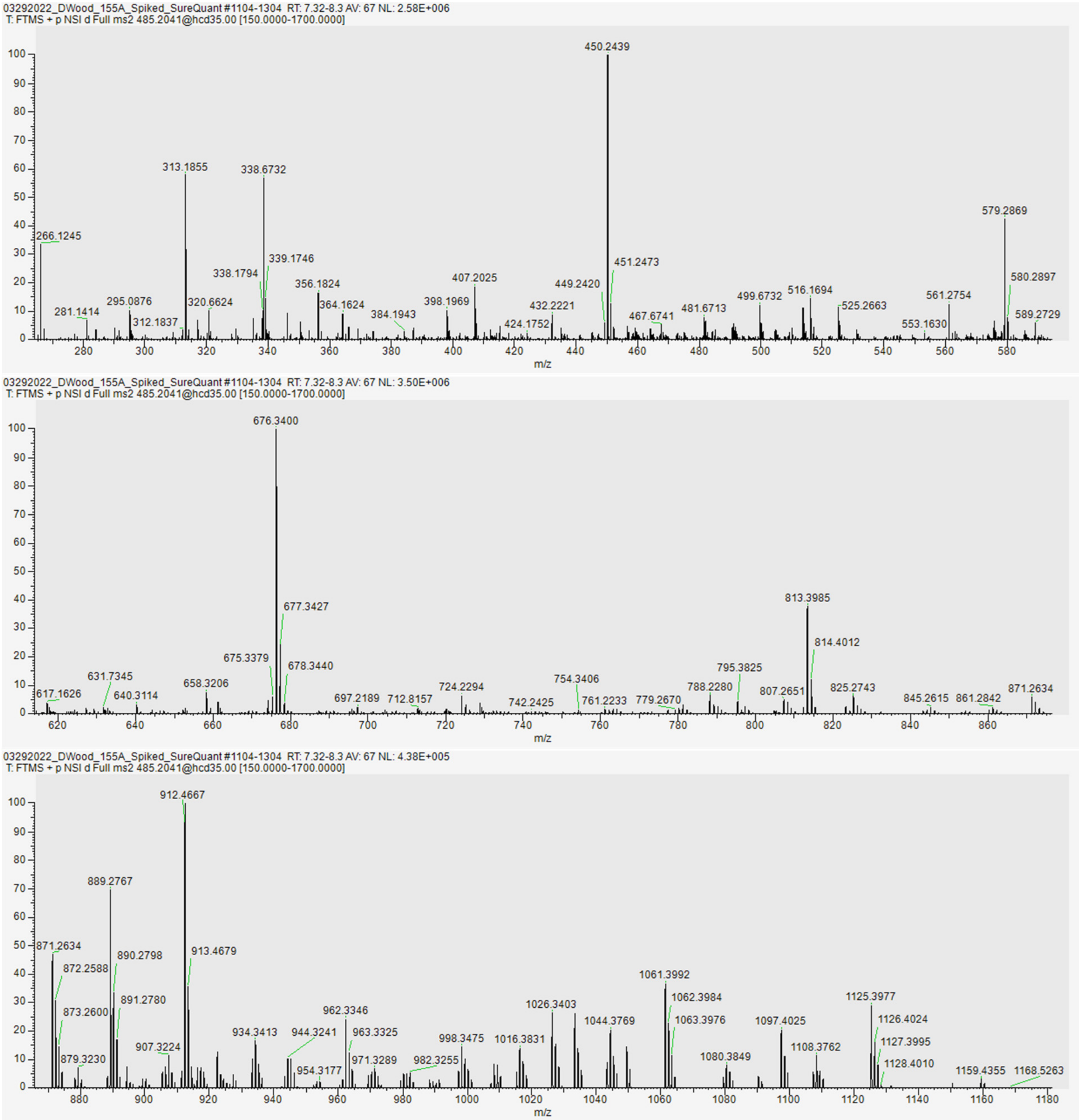

HQC(cys->dha)M(ox)C(sulfide)GNTHVHPEHAGR(Label:13C(6)15N(4))						
**MH^{+1}(av)**	**MH^{+1}(mono)**	**MH^{+2}(av)**	**MH^{+2}(mono)**	**MH^{+3}(av)**	**MH^{+3}(mono)**	**MH^{+4}(mono)**
1939.1	1937.8	970.04	969.4	647.03	646.6	485.2040
**b**	**b^{+2}**				**y**	**y^{+2}**
---	---	1	**H**	17	---	---
266.125	133.566	2	**Q**	16	1800.74	900.871
335.146	168.077	3	**C(cys->dha)**	15	1672.68	836.842
482.182	241.595	4	**M(ox)**	14	1603.66	802.331
617.163	309.085	5	**C(sulfide)**	13	1456.62	728.814
674.184	337.596	6	**G**	12	1321.64	661.323
788.227	394.617	7	**N**	11	1264.62	632.812
889.275	445.141	8	**T**	10	1150.57	575.791

1026.33	513.671	9	**H**	9	1049.53	525.267
1125.4	563.205	10	**V**	8	912.467	456.737
1262.46	631.734	11	**H**	7	813.399	407.203
1359.51	680.261	12	**P**	6	676.34	338.674
1488.56	744.782	13	**E**	5	579.287	290.147
1625.62	813.311	14	**H**	4	450.245	225.626
1696.65	848.83	15	**A**	3	313.186	157.097
1753.67	877.341	16	**G**	2	242.149	121.578
---	---	17	**R(Label:13C(6)15N(4))**	1	185.127	93.0672

Loss from M(ox)				b4	b5	b6	b7	b8	b9	b10	b11
b-$SOCH_4$	---	---	---	418.183	553.165	610.186	724.229	825.277	962.336	1061.4	1198.46

Rat NAc-Immunoprecipitated HDAC5 peptide: HQC(Cys->Dha)MC(Sulfide)GNTHVHPEHAGR

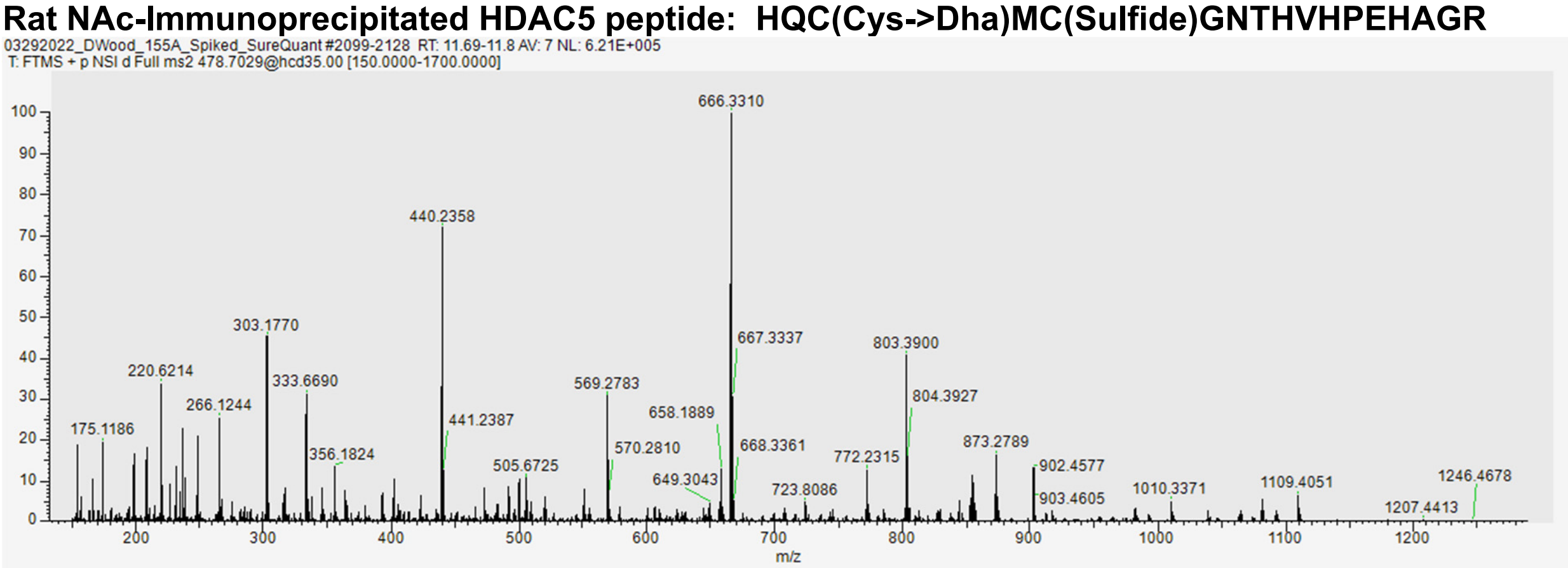

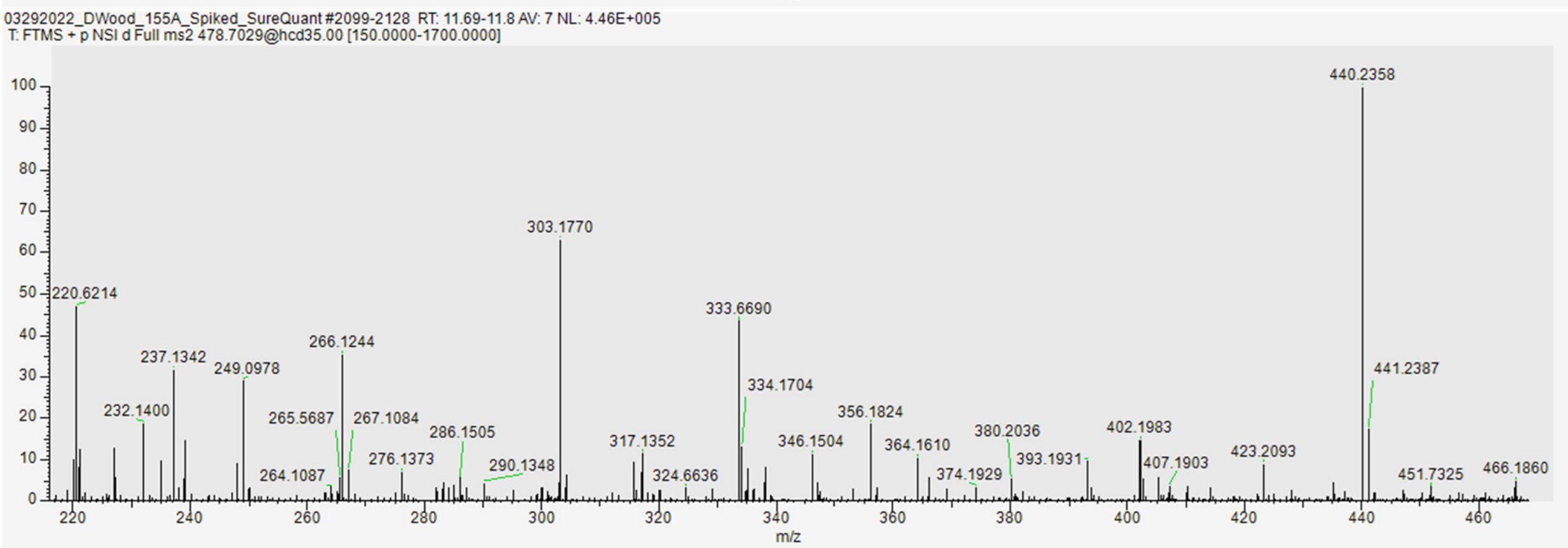

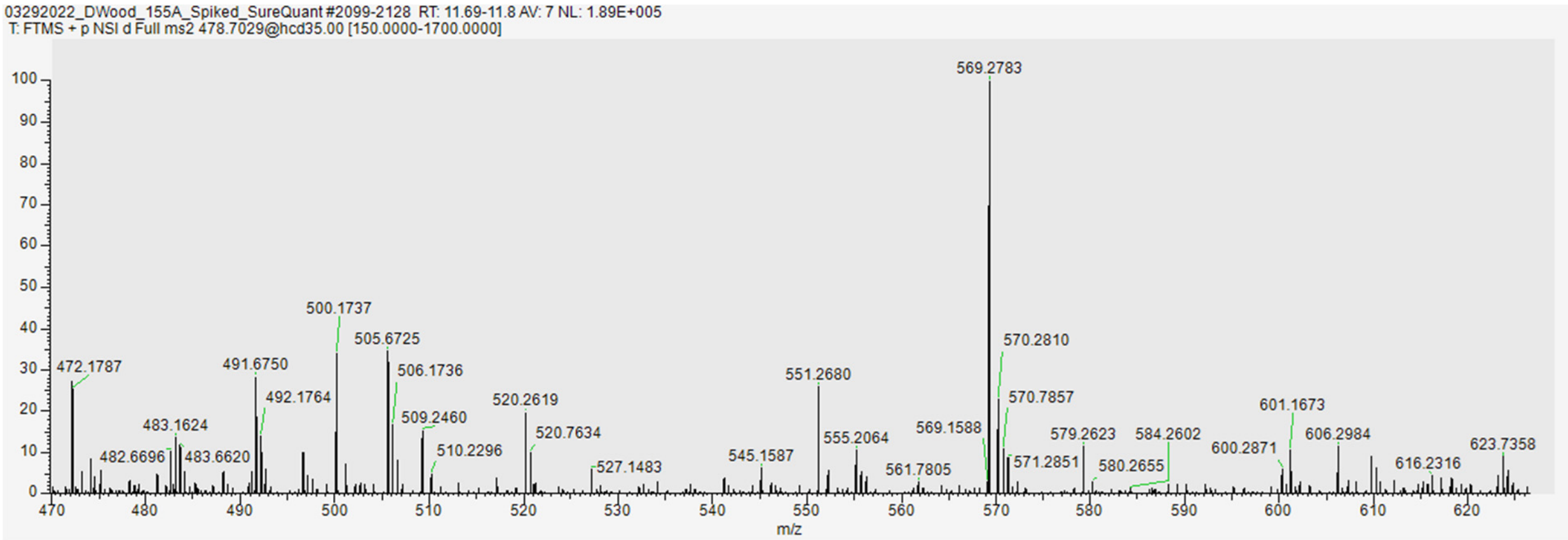

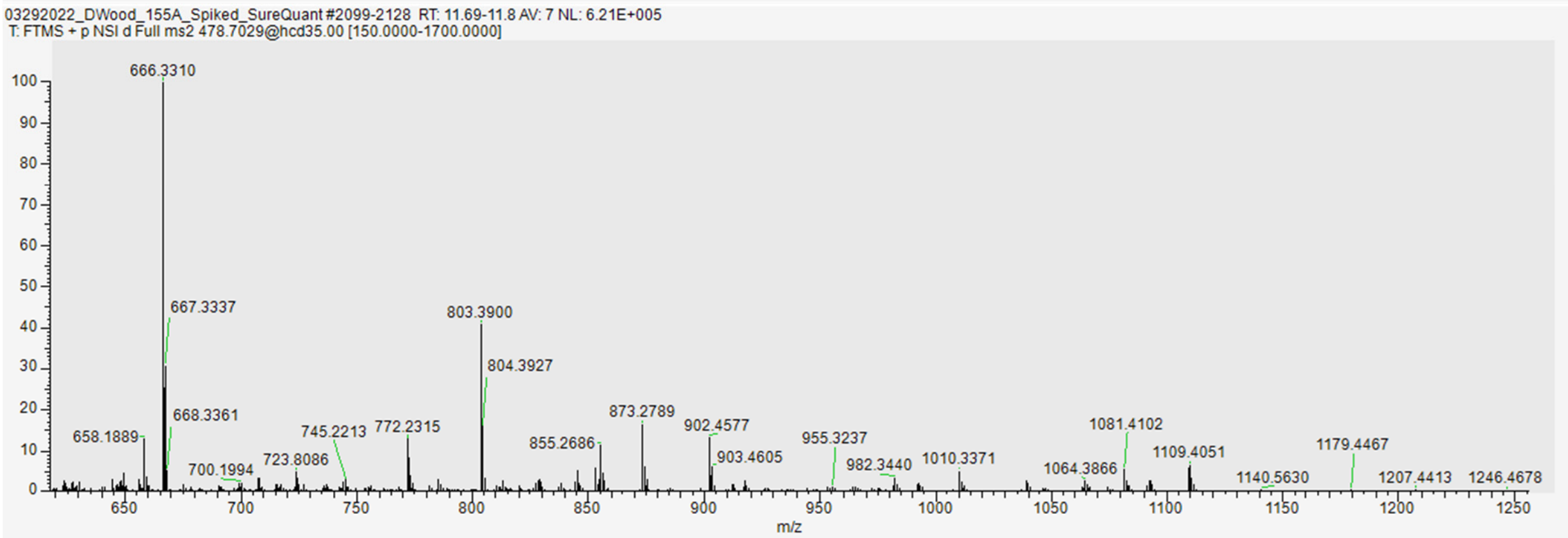

HQC(cys->dha)MC(sulfide)GNTHVHPEHAGR						
MH+1(av)	MH+1(mono)	MH+2(av)	MH+2(mono)	MH+3(av)	MH+3(mono)	MH+4(mono)
1913.1	1911.8	957.07	956.4	638.39	637.94	478.7032
**b**	**b+2**				**y**	**y+2**
---	---	1	**H**	17	---	---
266.125	133.566	2	**Q**	16	1774.73	887.87
335.146	168.077	3	**C(cys->dha)**	15	1646.67	823.84
466.187	233.597	4	**M**	14	1577.65	789.33
601.168	301.088	5	**C(sulfide)**	13	1446.61	723.809
658.189	329.598	6	**G**	12	1311.63	656.319
772.232	386.62	7	**N**	11	1254.61	627.808
873.28	437.144	8	**T**	10	1140.57	570.787
1010.34	505.673	9	**H**	9	1039.52	520.263
1109.41	555.207	10	**V**	8	902.459	451.733
1246.47	623.737	11	**H**	7	803.391	402.199
1343.52	672.263	12	**P**	6	666.332	333.67
1472.56	736.785	13	**E**	5	569.279	285.143
1609.62	805.314	14	**H**	4	440.236	220.622
1680.66	840.833	15	**A**	3	303.178	152.092
1737.68	869.343	16	**G**	2	232.14	116.574
---	---	17	**R**	1	175.119	88.0631

Heavy Labeled Synthetic peptide:
HQC(cys->dha)MC(sulfide)GNTHVHPEHAGR(Label:13C(6)15N(4))

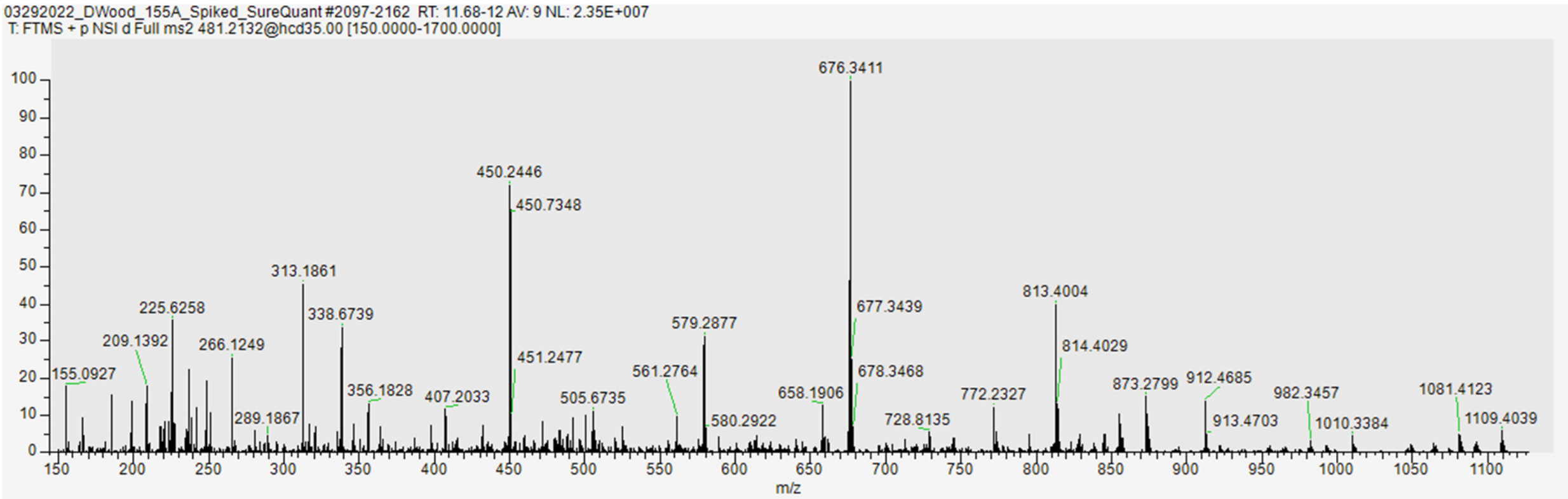

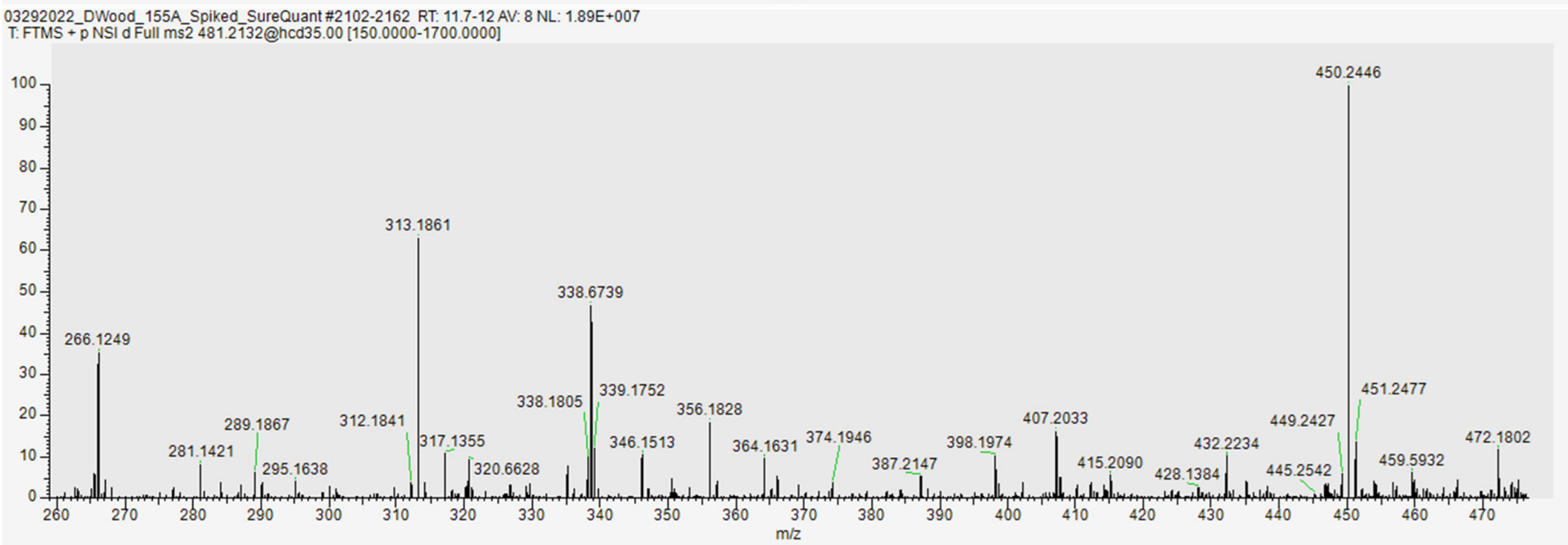

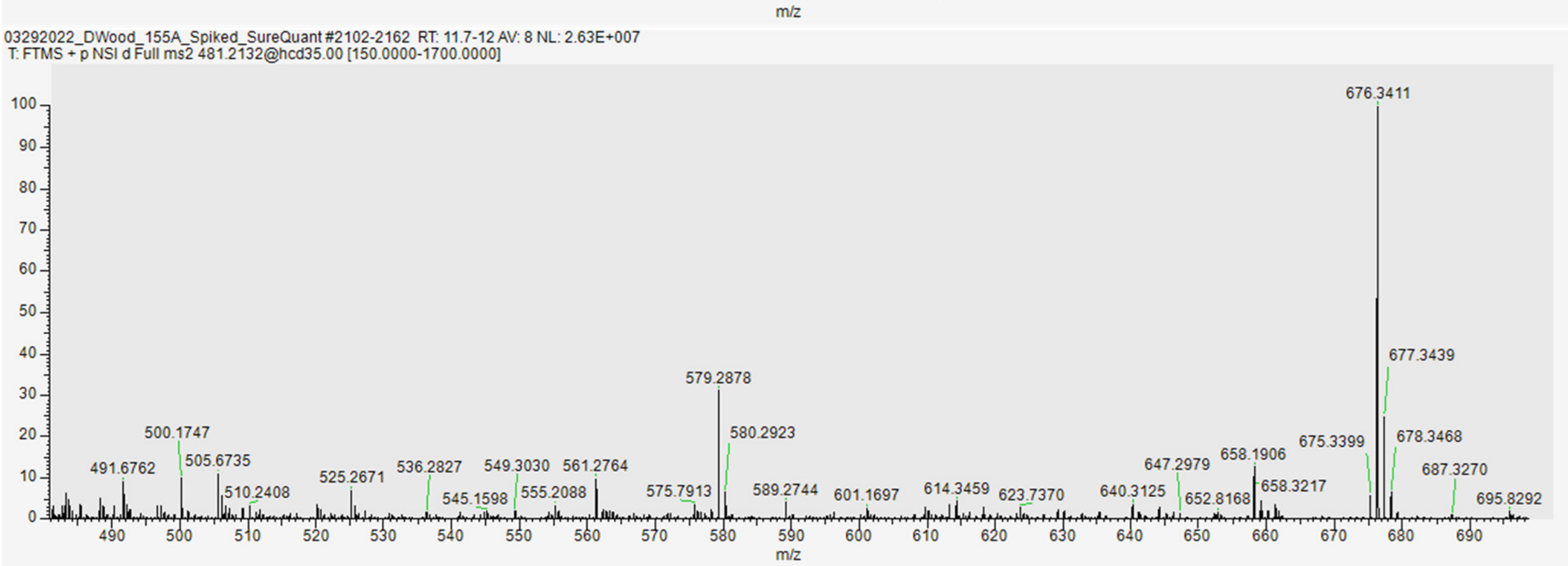

HQC(cys->dha)MC(sulfide)GNTHVHPEHAGR(Label:13C(6)15N(4))						
**MH^{+1}(av)**	**MH^{+1}(mono)**	**MH^{+2}(av)**	**MH^{+2}(mono)**	**MH^{+3}(av)**	**MH^{+3}(mono)**	**MH^{+4}(mono)**
1923.1	1921.8	962.04	961.4	641.69	641.27	481.2052
**b**	**b^{+2}**				**y**	**y^{+2}**
---	---	1	**H**	17	---	---
266.125	133.566	2	**Q**	16	1784.74	892.8737

335.146	168.077	3	**C(cys->dha)**	15	1656.68	828.8444
466.187	233.597	4	**M**	14	1587.66	794.3337
601.168	301.088	5	**C(sulfide)**	13	1456.62	728.8135
658.189	329.598	6	**G**	12	1321.64	661.3228
772.232	386.62	7	**N**	11	1264.62	632.8121
873.28	437.144	8	**T**	10	1150.57	575.7906
1010.34	505.673	9	**H**	9	1049.53	525.2668
1109.41	555.207	10	**V**	8	912.467	456.7373
1246.47	623.737	11	**H**	7	813.399	407.2031
1343.52	672.263	12	**P**	6	676.34	338.6737
1472.56	736.785	13	**E**	5	579.287	290.1473
1609.62	805.314	14	**H**	4	450.245	225.626
1680.66	840.833	15	**A**	3	313.186	157.0965
1737.68	869.343	16	**G**	2	242.149	121.578
---	---	17	**R(Label:13C(6)15N(4))**	1	185.127	93.0672

References for IS-PRM methodology:

1. Gallien S, Kim SY, Domon B. Large-Scale Targeted Proteomics Using Internal Standard Triggered-Parallel Reaction Monitoring (IS-PRM). Mol Cell Proteomics. 2015 Jun;14(6):1630-44. PMCID: PMC4458725.
2. Van Bentum M, Selbach M. An Introduction to Advanced Targeted Acquisition Methods. Mol Cell Proteomics. 2021;20:100165. PMCID: PMC8600983.

Supplemental Methods and Materials

Animals and Animal Care

Adult rats were singly housed in a climate-controlled environment (21 °C) on a 12-h light–dark cycle. Rats were male and female Long-Evans (HDAC5 experiments) or Sprague-Dawley (MEF2 and SCN4B experiments) wild-type rats (Charles River). All rats were habituated to the housing environment for at least 7 days prior to use in experiments and had food and water ad libitum except during the acquisition of cocaine self-administration as detailed below. All behavioral experiments were performed during the dark cycle as described below and were approved by the MUSC Institutional Animal Care and Use Committee in facilities accredited by the American Association for the Accreditation of Laboratory Animal Care. All procedures were conducted in accordance with the guidelines established by the NIH and the National Research Council.

Recombinant plasmids and viral vectors

N-terminal 3XFLAG tagged HDAC5 point mutants and c-terminal HA tagged HDAC3 were created via a PCR-based strategy using the previously described neurotropic adeno-associated vector serotype 2 (AAV2) HDAC5-WT, -3SA (1), and HDAC3-FLAG (Addgene #13819) vectors as templates. MEF2C-HA, FLAG-HDAC4, and MRE-mCherry plasmids were obtained from colleagues. HDAC5, HDAC3, MEF2C, and HDAC4 expression vectors used CMV as a promoter. *Mef2a* and *Mef2d* mRNA levels were reduced using the previously described AAV2 short hairpin RNA vectors (2). *Scn4b* mRNA levels were reduced using a AAV2 short hairpin RNA under to control of a U6 promoter (AAV2-shScn4b, sequence: GCGAAACATCCAGGATTCTCAT). Control viruses include AAV2-GFP (promoter: CMV) and AAV2-shLuciferase (sequence: CTTACGCTGAGTACTTCGA). All viruses were packaged by the UofSc viral vector core and had titers >1 X 10^{11} viral genomes/ul.

Stereotaxic Surgery

Stereotaxic surgery was performed under general anesthesia with isoflurane (induction 5% v/v, maintenance 1–2% v/v). Coordinates to target the NAc were +1.6 mm anterior, +2.8 mm lateral, and −7.1 mm ventral from bregma (relative to skull) at a 10° angle. Ketofen (MWI Animal Health, 5mg/kg, s.c.) was injected at the start of surgery for pain relief. Viruses were delivered bilaterally using Hamilton syringes at a rate of 0.05 µL every 30 seconds for a total 1 µL per hemisphere with an additional 10 minutes before needles were retracted. Viral placements were confirmed by FLAG signal for AAV-HDAC5 (all mutants) expression, by mCherry signal for AAV-shMef2d, and GFP signal for AAV-shMef2a, AAV-shScn4b, and AAV-shLuciferase.

Rat Intravenous Catheterization Surgery. Rats were anesthetized with isoflurane (induction 5% v/v, maintenance 1-2% v/v) and a chronic indwelling jugular vein catheter (SAI Infusion Technologies) was implanted. Ketofen (MWI Animal Health, 5mg/kg, s.c.) was injected at the start of surgery for pain relief and as needed for the following 3 days. Rats were allowed 3-5 days of recovery before starting behavioral experiments. Catheter patency was maintained by daily flushing with 0.1 ml cefazolin (MWI Animal Health), 0.1 ml heparin (Medline), and 0.1 ml Taurolidine-Citrate (TCS) lock solution (Access Technologies) until self-administration was completed. Catheter patency was checked with brevital sodium (Par Pharmaceutical) infusions the day preceding, and the last of, self-administration and as needed throughout the experiment. Rats with failed catheters either underwent a second catheterization surgery or were excluded from the study.

Rat Intravenous Cocaine and Sucrose Self-Administration. All self-administration experiments occurred in standard operant chambers with two retractable levers, a house light, a cue light, and speaker for cue tones, and a drug line connected to an external delivery pump (Med Associates). Rats were fasted one day before the start of behavioral experiments and held on a controlled diet (20g of food per day) to maintain 85-90% of ad libitum body weight throughout the course of active cocaine

self-administration to allow for lever baiting with lever extenders and/or food pellets as needed during the early phases of cocaine self-administration. Rats were then fed ad libitum during the subsequent phases of the experiment. During daily 2 hour sessions, rats were trained to press the active lever to receive a cocaine infusion (0.05 mL/infusion, 4mg/ml cocaine). During each infusion, a cue light was illuminated and a cue tone (2900 Hz) sounded, followed by a 20 second time out period during which subsequent active lever presses were registered but did not yield drug infusions or cues. Rats were run a fixed ratio (FR) schedule starting with FR1 followed by FR3 and FR5. In order to move from FR1 to FR3, rats must have had 5 days of 10 or more infusions; from FR3 to FR5 3 days of 10 or more infusions; and to complete FR5 4-5 days of 10 or more infusions. Following self-administration, rats were put in home-cage abstinence for one week before beginning extinction training. During extinction training, rats were placed back in the operant chambers for 2 hours. Active lever presses resulted in no drug infusions or light and sound cues. Rats received at least 6 days of extinction training until a criterion of less than 25 active lever presses for two consecutive days was met. Next, cue-induced reinstatement behavior was assessed on the following day during which rats were allowed to respond to light and sound cues without drug-infusion. Following at least 2 more extinction sessions and two consecutive days at less than 25 active lever presses, cocaine-primed reinstatement behavior was assessed by injecting a 10 mg/kg dose of cocaine before being placed in the operant chamber during which active lever presses did not result in drug infusion, light or sound cues.

Sucrose self-administration followed an identical schedule except did not undergo catheterization surgery or prime testing. Sucrose self-administration sessions were 1 hour long.

Immunohistochemistry was performed on all rats brains following the behavioral paradigm and viral placements were assessed by a blinded experimenter. Rats with inaccurate viral injections or with insufficient viral expression were excluded prior to data analysis.

Cell Culture

Human embryonic kidney cells (HEK293T) were cultured in Dulbeccos's modified Eagle medium (DMEM, Thermo Fisher) containing 10% (v/v) fetal bovine serum (Thermo Fisher) penicillin (50mg/ml)-streptomycin (50 units/ml; Millipore Sigma), and L-glutamine (4 mM; Sigma). Cultures were maintained by splitting at 1:10 and 1:20 dilutions every 3-5 days. Embryonic striatal neuron (E18/E19) cultures were taken from Long-Evans rats. Striatal tissues were digested with papain (10 unit/mL; Worthington) for 3 minutes at 37°C before the reaction was terminated. Tissue was then dissociated through passaging in a 10mL plastic pipette. Dissociated neurons were plated at 100,000 cells/well on cover glass precoated with Laminin and poly-D-lysine in a 24 well plate using DMEM supplemented with 10% (v/v) fetal bovine serum, penicillin-streptomycin penicillin (50mg/ml)-streptomycin (50 units/ml; Sigma), and L-glutamine (4 mM; Sigma) as culture medium. Cells were incubated for 24 hours in 5% CO_2 at 37°C in a humidified incubator before the culture media was changed to Neurobasal (Invitrogen) containing B27 supplement (2% v/v, Invitrogen), penicillin (50mg/ml)-streptomycin (50 units/ml; Sigma), and L-glutamine (4 mM; Sigma). On day *in vitro* 5, 30% of the culture media was replaced with fresh media.

Plasmid Transfection

HEK293T cells were plated at 100,000 cells/well (24 well plate), 200,000 cells/well (12 well plate), or 2 x 10^6 cells/plate (10 cm plate). After 24 hrs, cells were transfected using the calcium phosphate method using a total of 0.5 ug/well (24 well plate), 1 ug/well (12 well plate), or 10ug/plate (10 cm plate) of plasmid DNA. Cells were then incubated for 48 hours before harvesting for downstream application. Cultured striatal neurons were transfected using the calcium phosphate method on day *in vitro* 7 and allowed to incubate for 48 hours.

qPCR

Rats were briefly anaesthetized with isoflurane, decapitated, and the brain was removed. The brain was sectioned into 1 mm coronal slices and the NAc was obtained via tissue punch and flash frozen and stored at -80C. mRNA was extracted using QIAzol Lysis Reagent (Qiagen, cat: 56008534) and the RNeasy Micro Kit (Qiagen, cat: 74004). Copy deoxyribonucleic acid (cDNA) libraries were made with Superscript 3 kit (Invitrogen cat: 18080051). qPCR was performed using a Biorad CFX96 using the following primers:

scn4b Forward: 5'-GGGCTTTTGGGTCTCTTC-3'

scn4b Reverse: 5'-GAGGTTCTCAAAGCCATAACA-3'

mef2a Forward: 5'-TCAAGCCACACAACCTCTTG-3'

mef2a Reverse: 5'-CAGCATTCCAGGGGAAGTAA-3'

mef2d Forward: 5'-CAGCAGCCAGCACTACAGAG-3'

mef2d Reverse: 5'-GGCAGGGATGACCTTGTTTA-3'

beta-tubulin forward: 5'-CGACAATGAAGCCCTCTACGAC-3'

beta-tubulin reverse: 5'-ATGGTGGCAGACACAAGGTGGTTG-3'

3XFLAG-HDAC Immunoprecipitation

For experiments from cell culture (mass spectrometry, enzymatic activity, co-IP), cells were washed with ice cold phosphate buffered saline before being scraped off the plate in lysis buffer containing 50 mM Tris pH 6.8, 150 mM NaCl, 1% Triton X-100, 0.1% SDS, 0.5% Deoxycholate, 10 mM NaF, 0.1 mM Na_3VO_4, cOmplete Mini EDTA-free protease inhibitor tablet (Roche 11836170001). For experiments from tissue, rats were briefly anaesthetized with isoflurane and decapitated. The brain was extracted and the NAc obtained via tissue punch. Cells or tissue were then sonicated on ice for 15 seconds. The homogenates were then centrifuged at (16,000 RCF) at 4C for 15 minutes. The supernatant was then incubated with Anti-FLAG M2 (Millipore F2426) or Anti-HA (Millipore E6779) agarose beads for 2 hrs or

overnight with rotation at 4C. The protein-conjugated beads were then washed with lysis buffer and incubated with 2XSAB at 65C to elute proteins.

For the mass spectrometry experiments, the lysis buffer contained 40 mM n-ethylmaleimide (Sigma) to alkylate free cysteines. Following sonication, the homogenate was incubated at room temperature for 1 hour to allow alkylation to take place before proceeding. In Experiment 1 (Figure 1E), the protein-conjugated beads were incubated with 2XSAB containing 50 mM TCEP for 15 minutes at 65C and 45 minutes at room temperature to reduce unprotected cysteines before incubating with 10 mM Iodoacetamide (IAA, Thermo) for 30 minutes at room temperature to alkylate to newly reduced cysteines. We were unable to detect any IAA-conjugated (carbamidomethyl) cysteines, so the TCEP and IAA alkylation steps were removed for Experiment 2 (Figure 1E).

LC-MS/MS of HDAC proteins immunoprecipitated from HEK293 cells

Immunoprecipitated protein was separated by SDS-PAGE, stained with Coomassie blue, excised from the gel, and digested with porcine trypsin (Sigma). The resulting peptides were extracted, dried, desalted with C18 Zip Tips (Millipore), and dried under vacuum. Resolubilized peptides were analyzed by LC-MS/MS using an Easy-nLC 1200 LC system coupled to an Orbitrap Fusion Lumos (Thermo Scientific). Peptides were chromatographically separated with a 50 cm x 75 µm Acclaim PepMap RSLC C18 column (Thermo Scientific) using a gradient of 0-35% B in 180 minutes. Solvent A was 5% acetonitrile, 0.2% formic acid and solvent B was 80% acetonitrile, 0.2% formic acid. Data were acquired in data dependent acquisition mode with an inclusion list corresponding to CXC containing peptides with the following cysteine modifications: disulfide, N-ethylmaleimide (NEM), NEM+water, and/or carbamidomethyl. Precursor ions were fragmented by higher energy collisional dissociation (HCD) set at 35% collision energy. Precursor and fragment ions were detected in the Orbitrap at 60,000 and 15,000 resolution, respectively. The cycle time was 3 seconds and dynamic exclusion was

implemented. Precursor peptide ions not defined in the inclusion list were selected for fragmentation if the targets were not present or the cycle time permitted.

The raw data were searched using MaxQuant v. 2.0.1.0 (Max Planck Institute) using the following variable modifications for cysteine. To probe for an intramolecular disulfide bond within the CXC peptides, tandem mass spectra were searched for asymmetric fragmentation of the disulfide bond by HCD, yielding one cysteine that lost the sulfur (Cysteine (Cys) to Dehydroalanine (Dha) (-33.9878 Da) and one cysteine with an extra sulfur (Cys sulfide (+31.9721)). In addition, variable alkylation and oxidation of cysteines were included: n-ethyl maleimide (NEM) (+125.0477 Da), NEM + water (+143.0582 Da), mono oxidation (sulfenic) (+15.9949 Da), di oxidation (sulfinic) (+31.9898 Da), tri oxidation (sulfonic) (+47.9847 Da), and carbamidomethylation (+57.0215 Da). Methionine oxidation (+15.9949 Da) was also used as a variable modification. Data were searched against human FASTA sequences of HDAC4 and HDAC5 with a reversed decoy database and a false discovery rate of 1% at the PSM and peptide levels.

The peptide intensities reported in Fig. 1G are provided in Tables S1-S4. The results were filtered to include only the 10 most intense entries from the list. In general, these will coincide with the identifications with the highest MQ scores and the best spectrum quality.

LC-MS/MS of HDAC5 immunoprecipitated from NAc

HDAC5 protein was immunoprecipitated from the nucleus accumbens and prepared as described above. To confirm the presence of the disulfide linked form of the HDAC5 CMC peptide *in vivo*, a highly sensitive targeted, internal standard triggered-parallel reaction monitoring (IS-PRM) method was developed using the SureQuant acquisition mode on the Orbitrap Fusion Lumos MS (Thermo Scientific). Heavy, stable isotope-encoded peptides were synthesized by Vivitide (LubioScience GmbH, Zurich, Switzerland) and HPLC purified to > 95% purity. Due to the oxidization of methionine

detected in the HDAC5 CMC motif, peptides with an intramolecular disulfide linkage between cysteines 3 and 5 were synthesized with and without oxidized methionine at position 4 (HQCMCGNTHVHPEHAGR* (R* = Arginine ($^{13}C_6$, $^{15}N_4$)). The peptides, referred as PepM and PepMox, were provided as dried aliquots and stored at -80 °C. Stock solutions of each were first prepared at 1 pmol/µl in 2% acetonitrile, 0.2% formic acid, and further diluted prior to analysis. Peptides were analyzed by LC-MS/MS in "survey mode" using a gradient of 2-25%B in 60 min; 25% for 10 min; 25-90% in 5 min to determine the optimal precursor ions, product ions, and signal intensity to trigger data acquisition. Five, C-terminal y ion fragments (y^4-y^8) and a N-terminal (b^8) fragment ion were used to build the SureQuant acquisition method (supplemental data). Synthetic peptides were spiked into trypsin-digested HDAC5 isolated from the nucleus accumbens prior to LC-MS/MS analysis.

System Preparation for Molecular Dynamics

The initial model for the human HDAC5 catalytic domain (residues 680-1083) was obtained from the AlphaFold database (3). The positions of the Zn^{2+}, K^+, and Zn^{2+}-coordinating waters were obtained from the structure of HDAC4 (PDB ID: 5A2S) (4). For the ligand-bound state, one coordinating water was removed and the substrate peptide (H3K9, sequence TARK$_{ac}$ST) modeled based on the coordinates from a structure of HDAC8 (PDB ID: 2V5W) (5). Structures were minimized using ff14SB with included additional parameters for acetylated lysine in OpenMM, using custom restraints for the coordinated Zn^{2+} ions based on the structure of HDAC4 (PDB ID: 5A2S) (4,6,7). For disulfide bond systems, the structural Zn^{2+} ion was removed and the disulfide manually added between residues C696 and C698 prior to minimization. Protonation states for most residues were assigned using PROPKA, with the exception of C696 and C698, which were deprotonated or involved in a disulfide bond for the 2x Zn^{2+} and disulfide systems, respectively (8).

Molecular Dynamics Simulations

All simulations were performed using AMBER20 with the ff14SB forcefield with additional acetyllysine parameters (ff14SB_modAA) (7,9). The structures were solvated in a TIP3P water box with 10Å padding from the solute (10). All bonds to hydrogen were constrained using the SHAKE algorithm and the particle mesh Ewald method was used for long-range electrostatics (11,12). A constant temperature of 300K and constant pressure were maintained using Langevin dynamics and Berendsen's barostat, respectively (13,14). Neutralizing Na^+ and Cl^- ions were added to an approximate concentration of 0.15M NaCl. The parameters for simulation of the Zn^{2+} ions in both the catalytic and structural sites were included from the Lennard-Jones 12-6-4 potentials for divalent ions (15).

The solvent, Na^+, and Cl^- ions were first minimized by restraining the solute for 2700 steps of steepest descent and 300 steps of conjugate gradient minimization. The solute restraint was relaxed to 50 kcal mol^{-1} $Å^{-2}$, 20 kcal mol^{-1} $Å^{-2}$, and 5 kcal mol^{-1} $Å^{-2}$ for 8000 SD steps and 2000 CG steps per restraint value. The system was then heated from 0 K to 300 K in six steps of 50 K with a solute restraint of 5 kcal mol^{-1} $Å^{-2}$ under the NVT ensemble. The solute restraint was then reduced to 2, 0.1 and 0.05 kcal mol^{-1} $Å^{-2}$ for 8000 SD and 2000 CG steps per restraint value under the NVE ensemble. Following this, the system is equilibrated in NPT with restraints of 0.5, 0.1, 0.04, and 0.01 kcal mol^{-1} $Å^{-2}$ for 50, 100, 400, and 400ps. Finally, the system undergoes a 10ns unrestrained run with a 2fs timestep before the 250ns production run in the NPT ensemble. 100ns was discarded from the beginning of each run to avoid equilibration artifacts based on analysis of structural factors.

Two structural variables were examined via simulation: ligand/apo and 2x Zn^{2+}/disulfide. All permutations were considered, yielding a total of four systems. Each system was simulated in four independent runs of 260ns, totaling 640ns of production MD once equilibration time was considered per system. Trajectory analysis was performed using CPPTRAJ (16).

Deacetylase Activity Assay

A class IIa HDAC deacetylase activity kit (BPS Biosciences #50065). HEK 293T cells were transfected with 3XFLAG-HDAC5 constructs which were then immunoprecipitated as described above. The HDAC5-conjugated beads were then equilibrated with the kit assay buffer and successive volumes were loaded onto a black, low binding NUNC microtiter plate. The kit was then used as directed and fluorescence was measured using a SpectraMax M5 plate reader (Molecular Devices)

Western Blotting

Samples were homogenized as described above (see **immunoprecipitation)** combined with 2XSAB contained 10% beta-mercaptoethanol and incubated at 65C for 15 minutes. The samples were loaded into BioRad tris-glycine eXtended precast gels (7.5% or 4%-20% gradient) and run at 60V for 10 minutes and then 250V for 25 minutes. The gels were then transferred onto a BioRad Trans-Blot Turbo Transfer Pack (midi format, 0.20 μm polyvinylidene difluoride (PVDF), cat: 1704157) using the BioRad Trans-Blot Turbo Transfer System and run at 2.5 A, 25 V for 10 min and placed in Odyssey or Intercept blocking solution for 1 hour. The membrane was then incubated with primary antibodies: Mouse anti-FLAG M2 1:1000 (Sigma F1804), rabbit anti-HA 1:1000 (Sigma-Aldrich H6908), Chicken anti-GFP 1:1000 (Aves Labs GFP-1020) at 4C overnight. After washing 3X with TBS-T, the membrane was incubated with secondary antibodies: 800CW goat anti-mouse (cat: 926-32210, LI-COR, 1:20,000), 680RD goat anti-mouse (cat: 926-68070, LI-COR, 1:20,000), 800CW goat anti-rabbit (cat: 926-32211, LI-COR, 1:20,000), 680RD goat anti-rabbit (cat: 926-68071, LI-COR, 1:20,000), 800CW donkey anti-chicken (cat: 926-32218, LI-COR, 1:20,000) or 680RD donkey anti-chicken (cat: 926-68075, LI-COR, 1:20,000). Blots were imaged on a LI-COR Odyssey CLx and analyzed with ImageStudio software.

Immunohistochemistry

Rats were anaesthetized with ketamine (66 mg/kg)/xylazine (1.66 mg/kg) and perfused with 4% paraformaldehyde. The brains were then extracted and drop fixed in 4% PFA overnight at 4C. The

brains were then cryoprotected in 30% sucrose in 50 mL conical tubes at 4C until the brains sunk. Brains were sectioned at 40 uM on a microtome and stored in PBS with 0.2% sodium azide. Tissue was blocked in buffer (3% bovine serum albumen, 3% normal donkey serum, 3% normal goat serum, 0.3% Triton-X, 0.3% Tween-20 in phosphate-buffered saline (PBS)) for at least 1 hour and then incubated overnight at 4C in blocking buffer containing chicken anti-GFP 1:1000 (Aves GFP-1020), mouse anti-FLAG 1:1000 (Sigma F1804), and/or mouse anti-SCN4B clone N168/6 1:1000 (Antibodies Inc. #75-198). The following day tissue was washed with PBS and then incubated with blocking buffer containing donkey anti-chicken 488 1:500 (Jackson 703-545-155), donkey anti-mouse 594 1:500 (Jackson 715-585-150), and/or donkey anti-mouse 488 1:500 (Thermo A-21202). Tissue was then washed in PBS containing 1:5,000 concentration of bisbenzimide (Hoescht 33342, Invitrogen) for 10 minutes followed by two PBS washes. Tissue sections were then mounted onto microscope slides with Aquamount (Epredia 14-390-5) or Prolong Gold (Invitrogen P36930) mounting medium and imaged on a Leica Thunder 3-dimensional (3D) Tissue Imager. Images were processed with ImageJ.

Nuclear/Cytoplasmic Ratio

HDAC5 mutants were expressed in rat NAc for 3 weeks and FLAG staining was conducted as described above. All fluorescent cells in a 20x field were scored as nuclear, cytoplasmic, or both under blinded conditions.

MRE-Luciferase Assay

Striatal neurons were cultured as described above. They were transfected with MRE-mCherry (17), which expresses mCherry as a transcriptional read-out of MEF2 activity, eGFP as a transfection control, and the three HDAC5 mutant plasmids on DIV 7 or 8 using the calcium phosphate method. The cells were allowed to express for 48 hours before they were fixed (4% paraformaldehyde, 2% sucrose, PBS) for 20 minutes, washed 3X with PBS. Cells were then washed in PBS containing 1:5,000 concentration of bisbenzimide (Hoescht 33342, Invitrogen) for 15 minutes, washed 3X with PBS, and then mounted

on microscope slide with ProLong Gold (Invitrogen P36930). Every GFP-positive cell on each coverslip was then imaged at 40X. Images were then processed in ImageJ. A ROI was drawn around each cell and the integrated density in each channel was measured. The mCherry integrated density value was then normalized to the GFP value for each cell.

Electrophysiology

Animals were live-decapitated; the brain was removed from the skull and acute coronal slices (300-μm thickness) containing NAc were obtained using slicer Leica VT1200s. Slices were prepared in a semi-frozen artificial cerebrospinal fluid (ACSF) containing (in mM): 127 NaCl, 2.5 KCl, 1.2 Na2H2PO4, 24 NaHCO3, 11 D-glucose, 1.2 MgCl2, and 2.40 CaCl2, 0.4 Na-ascorbate (pH 7.4, 315-320 mOsm). Kynurenic acid (5 mM) was added to this solution to avoid overactivity of glutamatergic receptors during execution.

The prepared slices were transferred to ACSF without kynurenic acid and left to recover at 37°C for 30 minutes. Subsequently, they were transferred to room temperature ACSF for an additional 30 minutes of recovery before recording. All solutions were continually equilibrated with 95% O_2 and 5% CO_2.

Neurons on the slices were observed using infrared differential interference contrast optics (DIC/infrared optics). Virally infected cells in NAc were identified by emitting red fluorescence under green light and green fluorescence under blue light. Among genetically marked neurons the MSNs were distinguished from interneurons by their morphology (medium round cell), and confirmed by higher membrane potential (up to -65 to -85 mV), and spiking patterns in response to depolarizing current injection.

Unless stated otherwise, all electrophysiological experiments were performed in whole cell voltage clamp mode at -70 mV using borosilicate pipettes (4-6 MΩ) pulled using NARISHIGE puller

(NARISHIGE, PG10) from borosilicate tubing (Sutter Instruments) and filled by an internal solution containing (in mM): 140 $CsMetSO_4$, 5 KCl, 1 $MgCl_2$, 0.2 EGTA, 11 HEPES, 2 NaATP, 0.2 Na_2GTP and 0.1 $CaCl_2$, pH 7.2–7.4 (pH 7.2, 290–295 mOsm). All recording data were acquired and analyzed by amplifier AXOPATCH 200B (Axon Instruments), digitizer BNC2090 (X National instruments) and software AxoGraphX v.1.8.0, Clampfit v 8.0 (pClamp, Molecular devices) and MiniAnalysis Program v.6.0.9 (Synaptosoft). Data were filtered at 2 kHz by AXOPATCH 200B amplifier (Axon Instruments) and digitized at 10-20 kHz via AxoGraph v.1.7.0. software.

Action potentials were initiated by a series of depolarizing current pulses of increasing intensities (0-500 pA in 20 pA steps; 1 s duration at 0.2 Hz) and recorded in whole-cell current clamp mode with a holding transmembrane current clamped at -70 mV. The pipette solution consisted of a potassium-based internal solution containing (in mM): 145 KGlu, 5 KCl, 1 MgCl2, 0.2 EGTA, 11 HEPES, 2 NaATP, 0.2 Na2GTP, and 0.1 CaCl2, with a pH range of 7.2–7.4 and an osmolarity of 290–295 mOsm. Intrinsic excitability was assessed by plotting the number of action potentials against the level of depolarization for each pulse. Amplitude accommodation was calculated as the change in amplitude between the first and last spike of the current pulse. Time to last spike was defined as the time from the start of the current pulse to the peak of the last spike. Duration of Spiking was defined as the time from the peak of the first spike to the peak of the last spike of each current pulse.

Statistics

One-Way ANOVA, Two-Way Repeated Measures ANOVA, and Mixed Effects Model (Restricted Maximum Likelihood) followed by Tukey's, Sidak's, or Fisher's post-hoc comparisons were used when comparing more than two groups. Two groups were compared using t-tests. Before t-tests and One-Way ANOVAs, ROUT outlier tests (Q=0.5% or Q=1%) were conducted and detected outliers removed. Significance was considered $p < 0.05$. Because the study was not powered to detect sex differences,

statistic were not run on the data disaggregated by sex. All statistics were performed using GraphPad Prism. All statistical tests are reported in (Statistics Table)

References

1. Taniguchi M, Carreira MB, Cooper YA, Bobadilla AC, Heinsbroek JA, Koike N, *et al.* (2017): HDAC5 and Its Target Gene, Npas4, Function in the Nucleus Accumbens to Regulate Cocaine-Conditioned Behaviors. *Neuron* 96: 130-144.e6.
2. Pulipparacharuvil S, Renthal W, Hale CF, Taniguchi M, Xiao G, Kumar A, *et al.* (2008): Cocaine Regulates MEF2 to Control Synaptic and Behavioral Plasticity. *Neuron* 59: 621–633.
3. Jumper J, Evans R, Pritzel A, Green T, Figurnov M, Ronneberger O, *et al.* (2021): Highly accurate protein structure prediction with AlphaFold. *Nature* 596: 583–589.
4. Luckhurst CA, Breccia P, Stott AJ, Aziz O, Birch HL, Bürli RW, *et al.* (2016): Potent, Selective, and CNS-Penetrant Tetrasubstituted Cyclopropane Class IIa Histone Deacetylase (HDAC) Inhibitors. *ACS Med Chem Lett* 7: 34–39.
5. Vannini A, Volpari C, Gallinari P, Jones P, Mattu M, Carfí A, *et al.* (2007): Substrate binding to histone deacetylases as shown by the crystal structure of the HDAC8–substrate complex. *EMBO Rep* 8: 879–884.
6. Eastman P, Galvelis R, Peláez RP, Abreu CRA, Farr SE, Gallicchio E, *et al.* (2024): OpenMM 8: Molecular Dynamics Simulation with Machine Learning Potentials. *J Phys Chem B* 128: 109–116.
7. Maier JA, Martinez C, Kasavajhala K, Wickstrom L, Hauser KE, Simmerling C (2015): ff14SB: Improving the Accuracy of Protein Side Chain and Backbone Parameters from ff99SB. *J Chem Theory Comput* 11: 3696–3713.

8. Dolinsky TJ, Czodrowski P, Li H, Nielsen JE, Jensen JH, Klebe G, Baker NA (2007): PDB2PQR: Expanding and upgrading automated preparation of biomolecular structures for molecular simulations. *Nucleic Acids Res* 35: 522–525.

9. Case DA, Aktulga HM, Belfon K, Ben-Shalom I, Brozell SR, Cerutti DS, *et al.* (2021): *Amber 2021*. University of California, San Francisco.

10. Jorgensen WL, Chandrasekhar J, Madura JD, Impey RW, Klein ML (1983): Comparison of simple potential functions for simulating liquid water. *J Chem Phys* 79: 926–935.

11. Darden T, York D, Pedersen L (1993): Particle mesh Ewald: An N·log(N) method for Ewald sums in large systems. *J Chem Phys* 98: 10089–10092.

12. Ryckaert J-P, Ciccotti G, Berendsen HJC (1977): Numerical integration of the cartesian equations of motion of a system with constraints: molecular dynamics of n-alkanes. *J Comput Phys* 23: 327–341.

13. Berendsen HJC, Postma JPM, Van Gunsteren WF, Dinola A, Haak JR (1984): Molecular dynamics with coupling to an external bath. *J Chem Phys* 81: 3684–3690.

14. Wu X, Brooks BR (2003): Self-guided Langevin dynamics simulation method. *Chem Phys Lett* 381: 512–518.

15. Li P, Merz KM (2014): Taking into Account the Ion-Induced Dipole Interaction in the Nonbonded Model of Ions. *J Chem Theory Comput* 10: 289–297.

16. Roe DR, Cheatham TE (2013): PTRAJ and CPPTRAJ: Software for processing and analysis of molecular dynamics trajectory data. *J Chem Theory Comput* 9: 3084–3095.

17. Harrington AJ, Raissi A, Rajkovich K, Berto S, Kumar J, Molinaro G, *et al.* (n.d.): MEF2C regulates cortical inhibitory and excitatory synapses and behaviors relevant to neurodevelopmental disorders. *eLife* 5: e20059.
